# Supplementary material for: Spatiotemporal in vivo tracking of polyclonal human regulatory T cells (Tregs) reveals a role for innate immune cells in Treg transplant recruitment
Source: Mol Ther Methods Clin Dev. 2020 Dec 10;20:324–36. doi: 10.1016/j.omtm.2020.12.003 (PMC7811063; doi:10.1016/j.omtm.2020.12.003)
Supplement: Document S1. Supplemental Materials and Methods and Figures S1–S9 [file mmc1.pdf]

OMTM, Volume *20*

Supplemental Information

Spatiotemporal *in vivo* tracking of polyclonal human regulatory T cells (Tregs) reveals a role for innate immune cells in Treg transplant recruitment

Jacinta Jacob, Suchita Nadkarni, Alessia Volpe, Qi Peng, Sim L. Tung, Rosalind F. Hannen, Yasmin R. Mohseni, Cristiano Scotta, Federica M. Marelli-Berg, Robert I. Lechler, Lesley A. Smyth, Gilbert O. Fruhwirth, and Giovanna Lombardi

SUPPLEMENTARY INFORMATION

This supplement contains Supplementary Methods detailing lentivirus production, radiotracer uptake assays, flow cytometry and microscopy of cells, and the determination of detection limits of traceable Tregs. Moreover, it contains several Supplementary Figures, which are referred to in the main text.

Supplementary Methods

Lentivirus production. For lentivirus production we used HEK293T cells (ATCC, USA), which were cultured in DMEM supplemented with 10% (v/v) heat-inactivated fetal calf serum (FCS), 100 units/mL penicillin and streptomycin, and 2 mM *L*-glutamine. HEK293T cells were transfected with the target plasmid pLNT/SFFV NIS-GFP and the corresponding packaging plasmids pΔ8.91 and pVSV-G as previously described.[1] Virus-containing culture supernatant was harvested after 48h, concentrated four-fold using PEG-IT (System Biosciences) and stored at -80C until use.

Flow cytometric marker analysis. Cells were stained in phosphate-buffered saline (PBS) supplemented with 1%(v/v) FCS/5mM EDTA using fluorescently conjugated primary antibodies at concentrations ranging 2-5 μg/mL. Antibodies used were specific for CD4 (OKT4), FOXP3 (PCH101), HLA-A2 (BB7.2) (all from eBioscience); CD25 (clone 4E3 for assessment of Treg purity; clone 2A3 for all other experiments), CTLA-4 (BNI3) (all from BD Bioscience); CD127 (A019D5), CD62L (DREG-56), CLA (HECA-452) (all from BioLegend), and CCR4 (205410l; R&D Systems). Only live cells were analyzed with dead cells being excluded based on counterstaining with a near-infrared dye (LIVE/DEAD Fixable Near-IR Dead Cell Stain kit; Thermo Fisher). Intracellular staining was performed using the Fix/Perm kit (eBioscience). Data were acquired using a LSRFortessa II (BDBioscience) and analyzed using FlowJo v.7 (Tree Star).

Fluorescence microscopy of NIS-GFP$^+$ Tregs. NIS-GFP$^+$ Tregs were plated onto retronectin-coated translucent sterile 24-well dishes and subjected to life cell microscopy using a wide-field fluorescence microscope equipped with a filter cube suitable for GFP imaging (EVOS FL Cell Imaging System; Thermo Fisher, UK).

***In vitro* radio tracer uptake in NIS-GFP$^+$ Tregs.** To determine reporter gene function 10^6 cells (Tregs or NIS-GFP$^+$ Tregs) were transferred into Eppendorf tubes, washed with ice-cold PBS, and resuspended in 1 mL HBSS. 50kBq [^{99m}Tc]TcO_4^- was added and cells were incubated at 37°C for 30 min. To block NIS function, 10 µL of a 500 µM $NaClO_4$ solution was added 20 min prior to the assay. Cells were pelleted, supernatant was collected, and cells were washed three times with 1 mL PBS before being resuspended in growth medium for γ-counting and further culture. Cellular radiotracer uptake was determined by quantifying radioactivity in cells and pooled supernatant/wash solutions using a γ-counter (1282-Compugamma, LKB-Wallac) and calculated using Equ.S1 (wherein Cpm represents decay-corrected radioactivity counts per minute).

$$\%Radioactivity\ uptake = \frac{Cpm\ [Cells]}{Cpm\ [Cells] + Cpm\ [Supernatant\ and\ Washes]} \cdot 100 \qquad \text{(Equ.S1)}$$

For assays involving pre-labelling to subsequently determine radiolabeling effects *e.g.* phenotype, suppression etc., 10x10^6 NIS-GFP$^+$ Tregs were incubated as above with 3MBq [^{99m}Tc]TcO_4^- resulting in typical cellular radioactivity levels of 78.4 ± 11.5 mBq/cell.

Determination of the minimum number of NIS-positive cells detectable by nanoSPECT/CT. To determine the detection sensitivity of NIS-GFP$^+$ Tregs in the nanoSPECT/CT scanner (Bioscan/Mediso, Silver Upgrade), we prepared cell pellets consisting of pre-labeled NIS-positive (86 ± 5.7 mBq [^{99m}Tc]TcO_4^-/cell) and NIS-negative untransduced Tregs. The total cell number per pellet was kept constant at 10^6 cells. This procedure has previously been used to determine the detection limit of reporter gene expressing cells in preclinical SPECT and PET instruments.[2, 3] Briefly, cell mixtures were prepared in 50 µL PBS$^{+/+}$ in Eppendorf Tubes, pelleted and immediately scanned in the nanoSPECT/CT equipment for 45 min. Reconstructed images were analyzed using Vivoquant whereby radioactivity was measured in volumes of interest drawn with the help of CT information (tube walls as boundaries). Measurements were performed in triplicate. In line with standard analytical procedures, we defined the limit of detection (LOD) to be 3-times the standard deviation above background signals (Fig.S4).

Supplementary References

1. Volpe, A, Man, F, Lim, L, Khoshnevisan, A, Blower, J, Blower, PJ, and Fruhwirth, GO. **Radionuclide-fluorescence Reporter Gene Imaging to Track Tumor Progression in Rodent Tumor Models**. *J Vis Exp*. 2018; **133**: e57088.
2. Fruhwirth, GO, Diocou, S, Blower, PJ, Ng, T, and Mullen, GE. **A whole-body dual-modality radionuclide optical strategy for preclinical imaging of metastasis and heterogeneous treatment response in different microenvironments**. *J Nucl Med*. 2014; **55**: 686-694.
3. Diocou, S, Volpe, A, Jauregui-Osoro, M, Boudjemeline, M, Chuamsaamarkkee, K, Man, F, Blower, PJ, Ng, T, Mullen, GED, and Fruhwirth, GO. **[(18)F]tetrafluoroborate-PET/CT enables sensitive tumor and metastasis in vivo imaging in a sodium iodide symporter-expressing tumor model**. *Sci Rep*. 2017; **7**: 946.
4. Dohan, O, Portulano, C, Basquin, C, Reyna-Neyra, A, Amzel, LM, and Carrasco, N. **The Na+/I symporter (NIS) mediates electroneutral active transport of the environmental pollutant perchlorate**. *Proc Natl Acad Sci U S A*. 2007; **104**: 20250-20255.
5. Khoshnevisan, A, Chuamsaamarkkee, K, Boudjemeline, M, Jackson, A, Smith, GE, Gee, AD, Fruhwirth, GO, and Blower, PJ. **18F-Fluorosulfate for PET Imaging of the Sodium-Iodide Symporter: Synthesis and Biologic Evaluation In Vitro and In Vivo**. *J Nucl Med*. 2017; **58**: 156-161.
6. Portulano, C, Paroder-Belenitsky, M, and Carrasco, N. **The Na+/I- symporter (NIS): mechanism and medical impact**. *Endocr Rev*. 2014; **35**: 106-149.
7. Paroder-Belenitsky, M, Maestas, MJ, Dohan, O, Nicola, JP, Reyna-Neyra, A, Follenzi, A, Dadachova, E, Eskandari, S, Amzel, LM, and Carrasco, N. **Mechanism of anion selectivity and stoichiometry of the Na+/I- symporter (NIS)**. *Proc Natl Acad Sci U S A*. 2011; **108**: 17933-17938.
8. Ashmore-Harris, C, Blackford, SJ, Grimsdell, B, Kurtys, E, Glatz, MC, Rashid, TS, and Fruhwirth, GO. **Reporter gene-engineering of human induced pluripotent stem cells during differentiation renders in vivo traceable hepatocyte-like cells accessible**. *Stem Cell Res*. 2019; **41**: 101599.
9. Man, F, Lim, L, Volpe, A, Gabizon, A, Shmeeda, H, Draper, B, Parente-Pereira, AC, Maher, J, Blower, PJ, Fruhwirth, GO*, et al.* **In Vivo PET Tracking of (89)Zr-Labeled Vgamma9Vdelta2 T Cells to Mouse Xenograft Breast Tumors Activated with Liposomal Alendronate**. *Molecular therapy : the journal of the American Society of Gene Therapy*. 2019; **27**: 219-229.
10. Volpe, A, Lang, C, Lim, L, Man, F, Kurtys, E, Ashmore-Harris, C, Johnson, P, Skourti, E, de Rosales, RTM, and Fruhwirth, GO. **Spatiotemporal PET Imaging Reveals Differences in CAR-T Tumor Retention in Triple-Negative Breast Cancer Models**. *Molecular therapy : the journal of the American Society of Gene Therapy*. 2020.

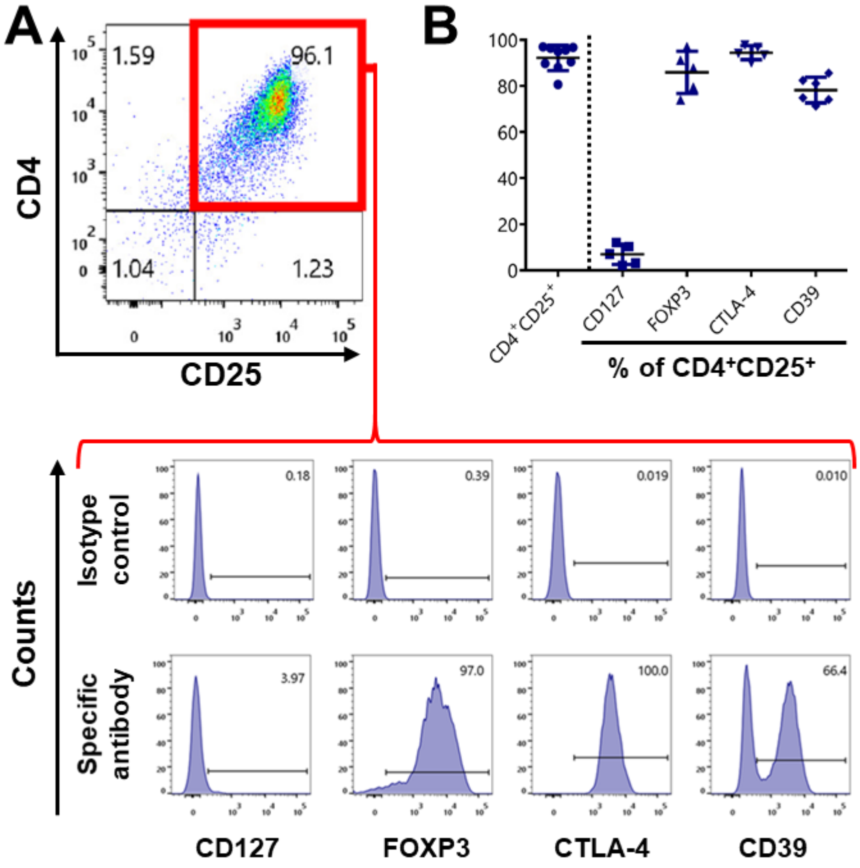

Figure S1. Quality of *in vitro* expanded human Tregs.| (A) Tregs were isolated from different human donors and expanded *in vitro* for 20 days. $CD4^+CD25^+$ population was analyzed for expression of CD127, FOXP3, CTLA-4 and CD39. **(B)** Cumulative data ($n \geq 5$) from individual donors indicating agreement with the expected phenotype for human Tregs; error bars represent SD.

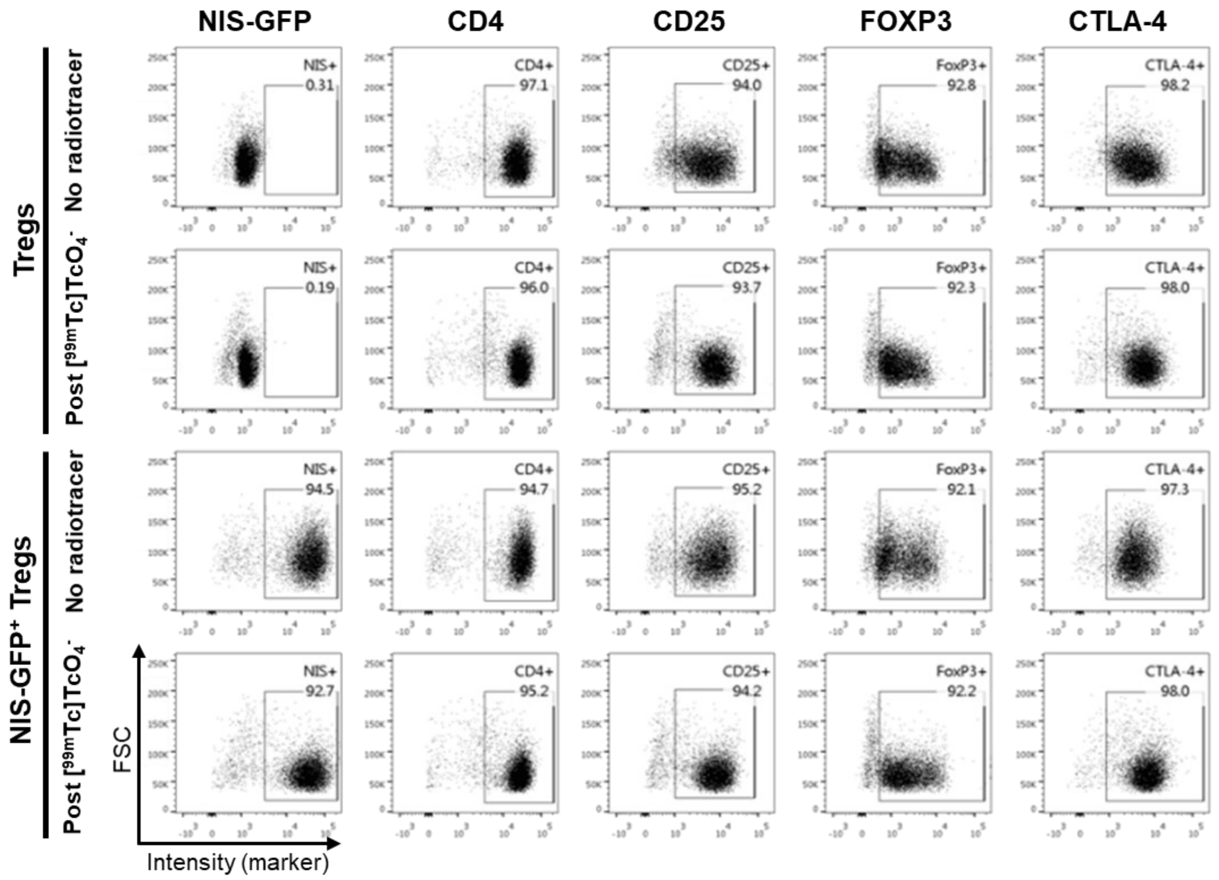

Figure S2. Representative individual data supplementing the marker panel analyses of Fig.2A-B.| Established Treg markers CD25, FOXP3 and CTLA-4 were quantified alongside CD4 (all *via* antibody staining) and NIS-GFP (*via* intrinsic GFP fluorescence). Tregs were analyzed 6 h (one half-life) after exposure to the NIS radiotracer [^{99m}Tc]TcO_4^-. Cumulative data describing the percentage and the mean fluorescence intensities of positive cells are shown in Fig.2A-B. Corresponding representative individual data are shown here.

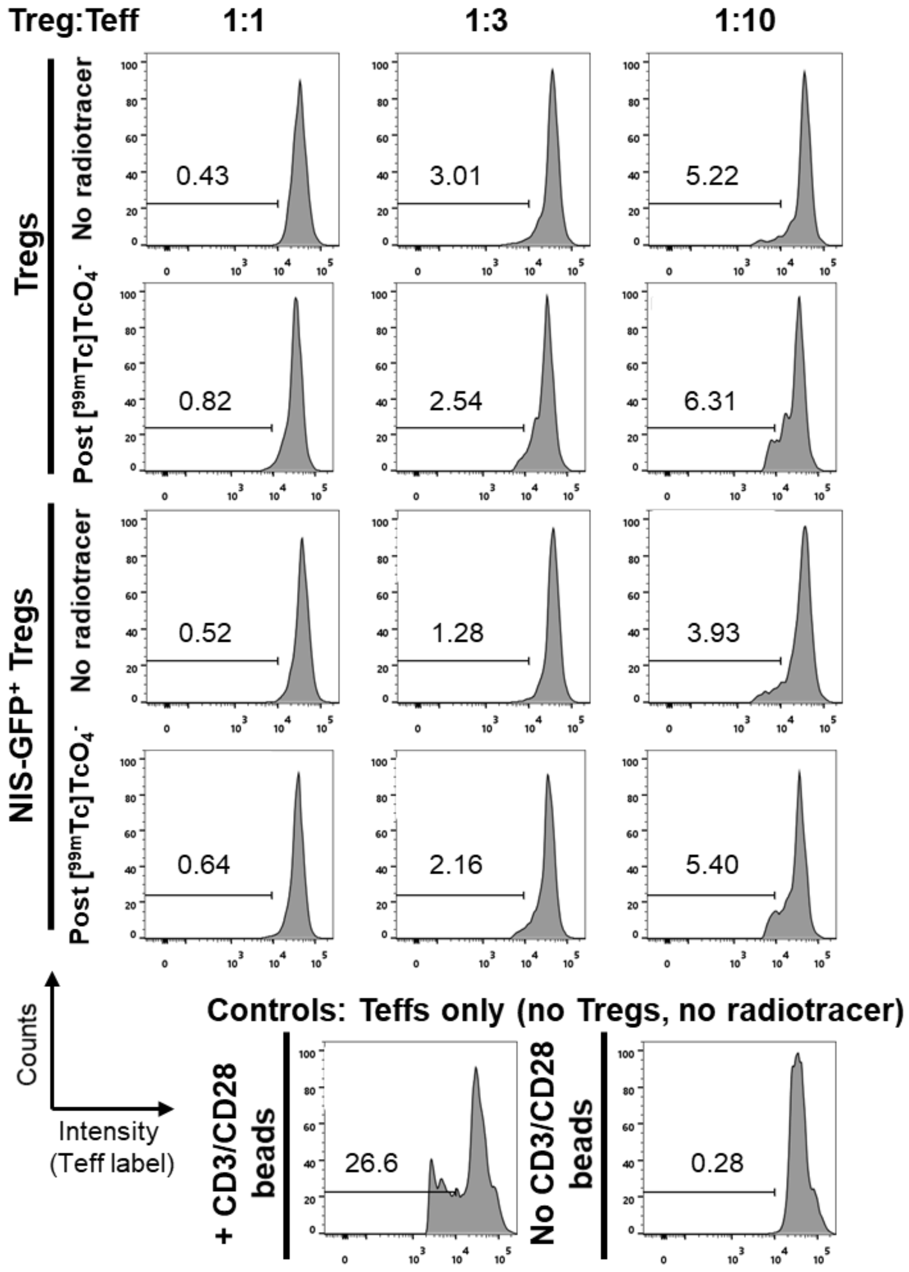

Figure S3. Representative individual data supplementing the Treg suppression analyses of Fig.2C.| Comparison of suppressive capacity of NIS-GFP$^+$ Tregs and untransduced Tregs in the presence of absence of radioactivity as indicated. Inset numbers are absolute %Teffs that proliferated in the presence of CD3/CD28 beads with headers indicating the relative Treg:Teff ratios. Teffs cultured in the absence of Tregs proliferated readily (left bottom) and served for data normalization; unstimulated Teffs did not proliferate (right bottom). Representative data of *n*=3 experiments (*n* different donors). Cumulative data are shown in Fig.2C.

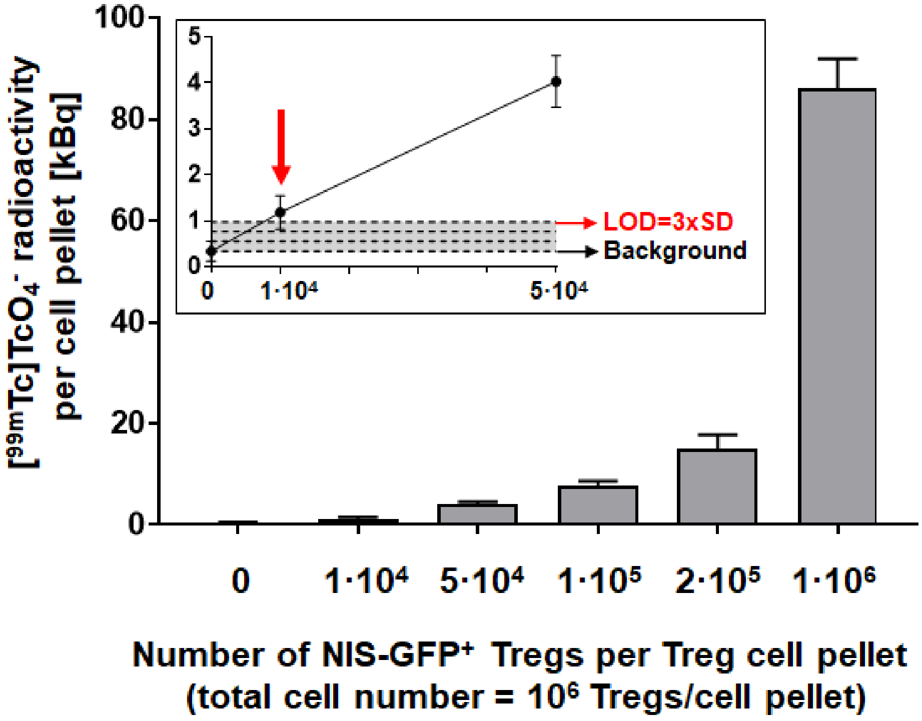

Figure S4. Determination of the detection sensitivity of NIS-GFP$^+$ Tregs.| NIS-GFP$^+$ Tregs were labeled with the radiotracer $^{99m}TcO_4^-$ and serially diluted into untransduced and unlabeled Tregs. Cell pellets in Eppendorf tubes were imaged and quantified using nanoSPECT (see Supplementary Methods). From triplicate measurements (error bars are SD), we determined the detection limit to be ~10,000 NIS-positive Tregs within a cell pellet of 10^6 untransduced Tregs.

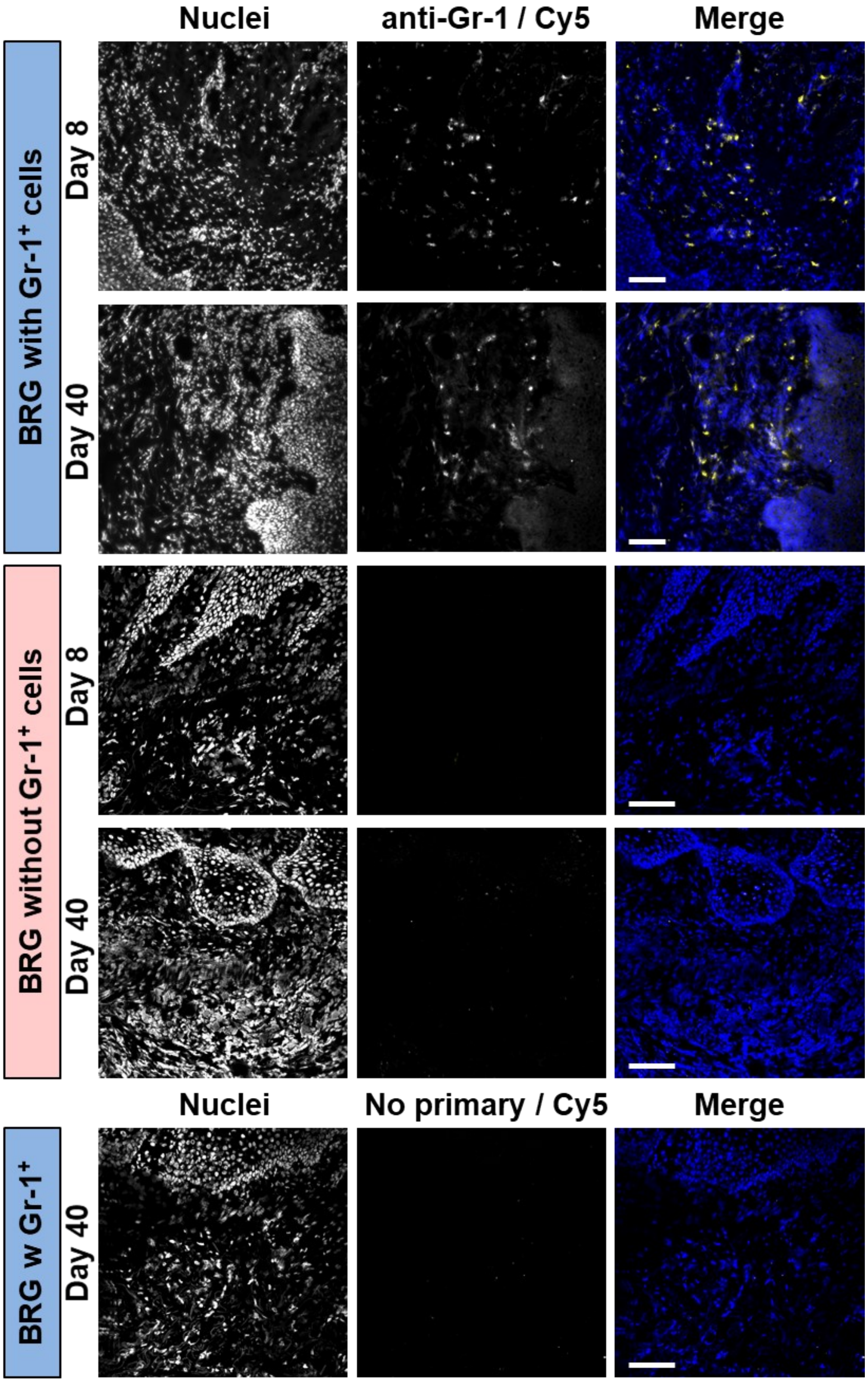

Figure S5 Treatment with anti-Gr-1 antibody depletes Gr-1^{+} cells *in vivo.*| Confocal micrographs of representative human graft sections of animals treated according to the scheme in Fig.4A (day 40; stained as indicated) on the indicated days; scale bars are 100 µm.

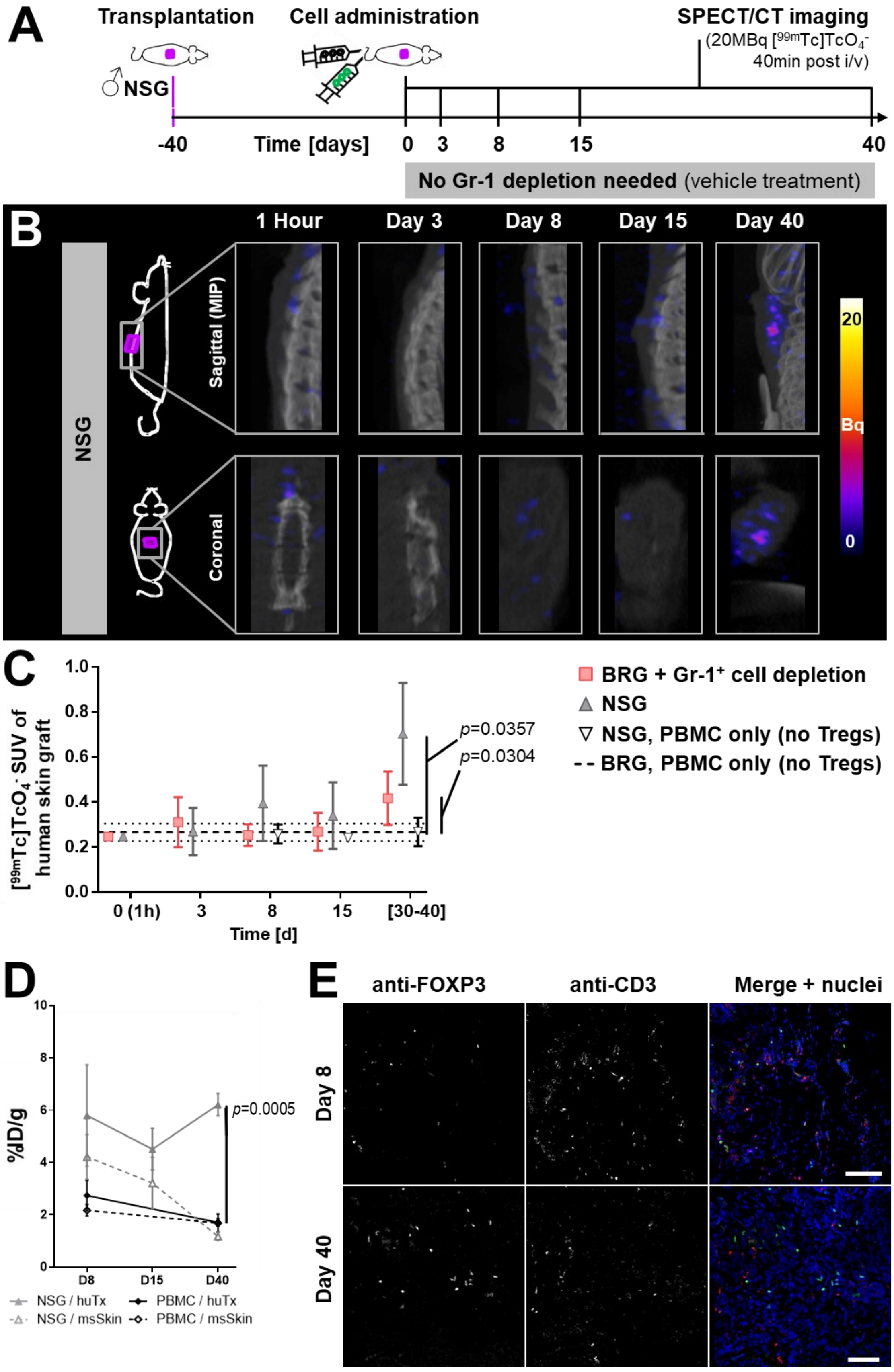

A
Transplantation
Cell administration
SPECT/CT imaging
(20MBq [99mTc]TcO4- 40min post i/v)
♂NSG
-40
Time [days]
0
3
8
15
40
No Gr-1 depletion needed (vehicle treatment)
B
1 Hour
Day 3
Day 8
Day 15
Day 40
NSG
Sagital (MIP)
Coronal
20
Bq
0
C
[99mTc]TcO4- SUV of human skin graft
Time [d]
0 (1h)
3
8
15
[30-40]
p=0.0357
p=0.0304
BRG + Gr-1+ cell depletion
NSG
NSG, PBMC only (no Tregs)
BRG, PBMC only (no Tregs)
D
%ID/g
D8
D15
D40
p=0.0005
NSG / huTx
NSG / msSkin
PBMC / huTx
PBMC / msSkin
E
anti-FOXP3
anti-CD3
Merge + nuclei
Day 8
Day 40

Figure S6. ***In vivo*** **tracking of NIS-GFP$^+$ Treg arrival at human skin grafts in NSG mice.| (A)** Experimental design: purple=human skin graft, green syringe = 5x10^6 NIS-GFP$^+$ Treg, black syringe = 5x10^6 PBMCs. **(B)** Longitudinal SPECT/CT imaging data of transplant-bearing animals (randomization upon adoptive cell transfer) at indicated time points post adoptive cell transfer. Images acquired and presented as in Fig.4; all images are on the same scale. Radioactivity above background indicates NIS-GFP$^+$ Treg presence. Representative images from the same animal. **(C)** Quantitative SUV analysis of imaging data. Cumulative data of all experiments (n=4); error bars represent SD; multiple t-tests with Holm-Sidak correction for multiple comparisons, p-values shown if < 0.05. For comparison data from Gr-1$^+$ cell-depleted BRG mice are shown (red). **(D)** *Ex vivo* radioactivity analysis of harvested tissues. Mouse skin was harvested from a region above lower spine. Human skin grafts were compared to mouse skin from the same animal using paired t-tests. No Treg/PBMC only control: not significant over all time points (p=0.2526), solid *vs.* dashed black lines; NSG: not significant for early time points (p>0.05) but significant on day 40 (p=0.0020), solid *vs.* dashed gray lines. Human skin grafts from NSG cohorts were compared by ANOVA on day 40 with control mice demonstrating a significant difference (p=0.0005), while mouse tissues did not differ between cohorts (A). Cumulative data; n=4 for days 8 and 40 and n=2 for day 15. **(E)** Immunofluorescence staining of harvested tissues. Indicated times refer to days after adoptive Treg transfer. Representative micrographs of NSG mice that received PBMC and NIS-GFP$^+$ Tregs. CD3 staining is indicative of PBMC-derived human T cells and Tregs, while FOXP3 staining identified human Tregs. Scale bars are 100 µm. Image-based quantification and *ex vivo* confirmation demonstrated a time course of NIS-GFP$^+$ Tregs signals at the transplants comparable to what we had observed for BRG mice with Gr-1$^+$ cell depletion (Fig.4-5).

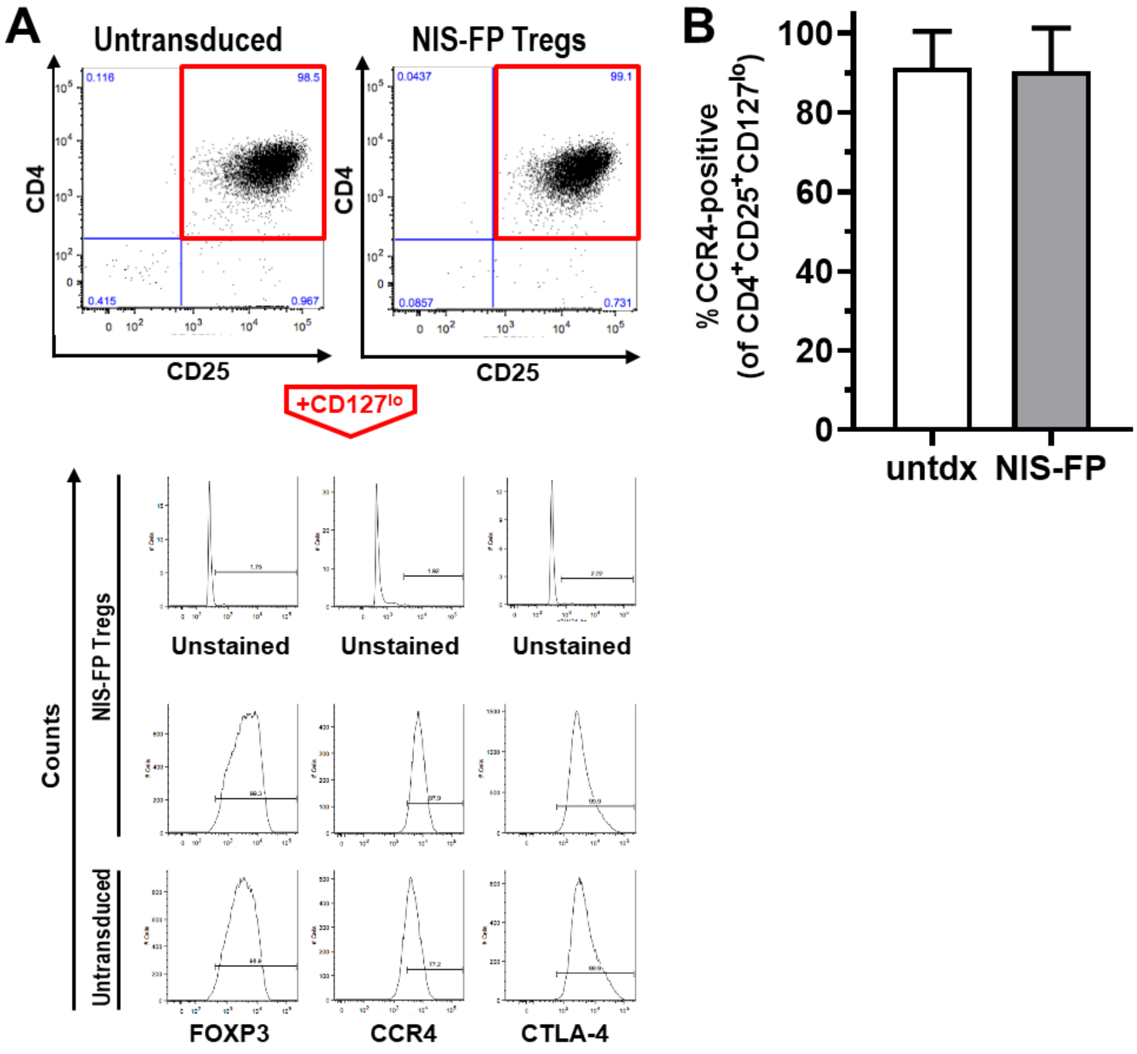

Figure S7. GMP-isolated and *in vitro* expanded Tregs retain their CCR4 expression upon transduction with the reporter gene NIS-FP.| (A) Tregs were isolated from different human donors and expanded *in vitro* for 20 days. $CD4^{+}CD25^{+}CD127^{low}$ Tregs were analyzed for FOXP3, CCR4 and CTLA-4 expression. Representative flow cytometry data are shown as histograms. **(B)** Cumulative data (n=5 individual donors) demonstrating no differences between untransduced and reporter-expressing Tregs (Student's *t*-test; p=0.8903); error bars represent SD.

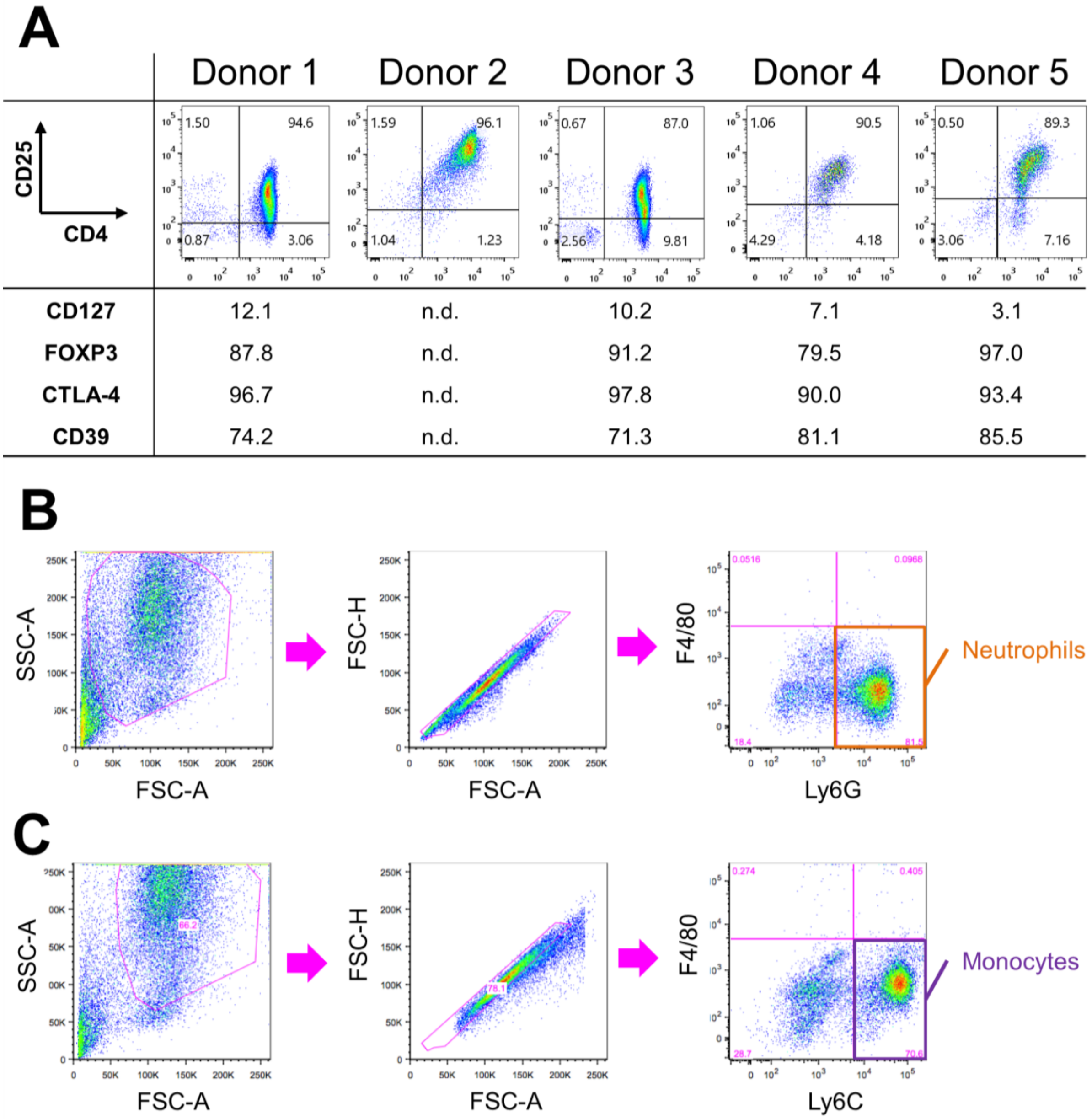

Figure S8. Input quality control data corresponding to purified cells used for experiments shown in Fig.7.| (A) Tregs isolated from individual human donors were analyzed for indicated markers by flow cytometry (numbers indicate percent expressing cells). Enrichment of murine **(B)** neutrophils and **(C)** monocytes.

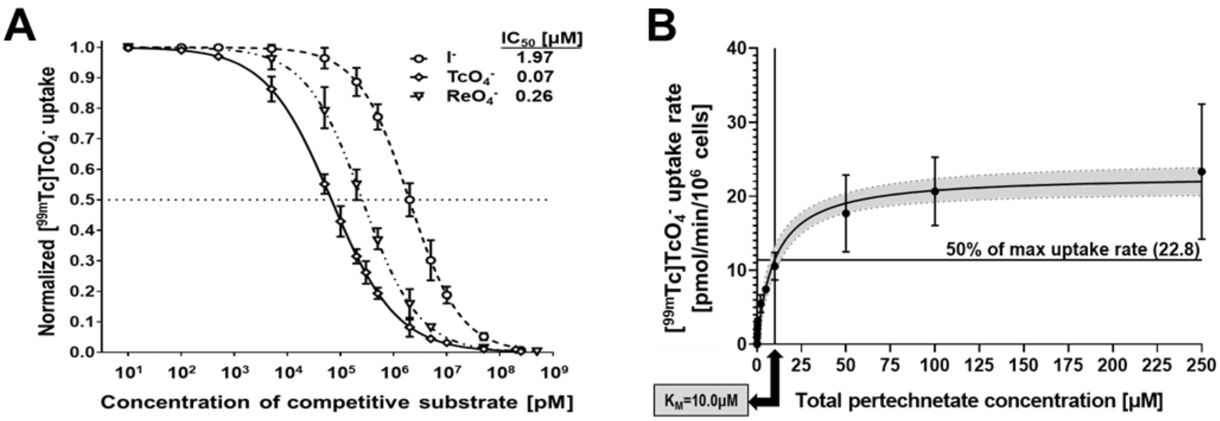

Figure S9. Substrate transport of NIS fused to a fluorescent protein. | (A) NIS anion transport was determined using a substrate competition assay with radioactive $[^{99m}Tc]TcO_4^-$ as the detectable trace anion. Briefly, epithelial breast cancer cells stably expressing NIS-mTagRFP[2] were incubated with 50 kBq/mL $[^{99m}Tc]TcO_4^-$ together with varying concentrations of the indicated anions, which were either non-radioactive I^- and ReO_4^- or the weakly radioactive beta emitter anion $[^{99}Tc]TcO_4^-$ (^{99}Tc radiation did not interfere with γ-emission measurements of ^{99m}Tc). The pertechnetate curve (solid) represents the reference measurement with the data for I^- and ReO_4^- indicating less efficient uptake of these substrates by NIS-mTagRFP compared to pertechnetate. This data is in agreement with data obtained by others using electrophysiological measurements of NIS without C-terminal tail modifications.[4] **(B)** As ^{99m}Tc decays to ^{99}Tc, reliable transport kinetic measurements using the radioactive substrate approach are possible, because the chemical species remains the same despite radioisotope decay and thus permitting constant substrate concentrations throughout the assay. In this way, we used the Michaelis-Menten model to model radiotracer uptake, which was previously shown to be valid for this this process. We determined the Michaelis-Menten constant of NIS-mTagRFP for the transport of the tetrahedral anion pertechnetate to be 10.0 µM (with a 95% confidence interval of [6.4 to 15.6] µM using non-linear regression with least-squares fitting). Pertechnetate is identical in charge and structure, and very similar in its anionic volume compared to ReO_4^-;[5] pertechnetate is also transported by NIS in an electroneutral manner like perrhenate.[6] Our data for the NIS-fusion protein are very close to what was previously reported for the Michaelis-Menten constant of the very similar NIS substrate ReO_4^- using unmodified NIS in an electrophysiological assay (3.5 µM;[7]). All error bars in (A) and (B) represent SD.

While we did not directly compare NIS with a NIS-fluorescent protein fusion in the same cells, we believe our data are sufficiently in agreement with previously reported data on unmodified NIS to suggest that for the purpose of *in vivo* cell tracking the NIS-fluorescent protein fusion reporter is a justified choice. Moreover, the NIS fluorescent protein fusion reporter provides added value by simplifying preclinical experimentation while not negatively impacting on NIS reporter performance. Moreover, the NIS-fluorescent protein reporters have been successfully used for *in vivo* cell tracking in various settings including many cancer cells[1-3, 8, 9], chimeric antigen receptor T-cells[10], and induced pluripotent stem cell-derived hepatocyte-like cells.[8]

<<<End of Supplementary Information>>>
