# Supplementary material for: Spatiotemporal in vivo tracking of polyclonal human regulatory T cells (Tregs) reveals a role for innate immune cells in Treg transplant recruitment
Source: Mol Ther Methods Clin Dev. 2020 Dec 10;20:324–36. doi: 10.1016/j.omtm.2020.12.003 (PMC7811063; doi:10.1016/j.omtm.2020.12.003)
Supplement: Document S2. Article plus supplemental information [file mmc2.pdf]

Molecular Therapy
Methods & Clinical Development
Original Article

Spatiotemporal *in vivo* tracking of polyclonal human regulatory T cells (Tregs) reveals a role for innate immune cells in Treg transplant recruitment

Jacinta Jacob,[1] Suchita Nadkarni,[2] Alessia Volpe,[3,6] Qi Peng,[1] Sim L. Tung,[1] Rosalind F. Hannen,[2] Yasmin R. Mohseni,[1,3] Cristiano Scotta,[1] Federica M. Marelli-Berg,[4] Robert I. Lechler,[1] Lesley A. Smyth,[1,5,7] Gilbert O. Fruhwirth,[3,7] and Giovanna Lombardi[1,7]

[1]MRC Centre for Transplantation, Peter Gorer Department of Immunobiology, School of Immunology and Microbial Science, King's College London, Guy's Hospital, London SE1 9RT, UK; [2]Centre for Cell Biology & Cutaneous Research, The Blizard Institute, Bart's and The London School of Medicine and Dentistry, Queen Mary University of London, London E1 2AT, UK; [3]Imaging Therapies and Cancer Group, School of Biomedical Engineering and Imaging Sciences, King's College London, London SE1 7EH, UK; [4]William Harvey Research Institute, Bart's and The London School of Medicine, Queen Mary University of London, London EC1M 6BQ, UK; [5]School of Health, Sport and Bioscience, Stratford Campus, University of East London, London E16 2RD, UK

Regulatory T cells (Tregs) are emerging as a new cell-based therapy in solid organ transplantation. Adoptive transfer of Tregs has been shown preclinically to protect from graft rejection, and the safety of Treg therapy has been demonstrated in clinical trials. Despite these successes, the *in vivo* distribution and persistence of adoptively transferred Tregs remained elusive, which hampers clinical translation. Here we isolated human Tregs using a GMP-compatible protocol and lentivirally transduced them with the human sodium iodide symporter to render them traceable *in vivo* by radionuclide imaging. Engineered human Tregs were characterized for phenotype, survival, suppressive capacity, and reporter function. To study their trafficking behavior, they were subsequently administered to humanized mice with human skin transplants. Traceable Tregs were quantified in skin grafts by non-invasive nano-single-photon emission computed tomography (nano-SPECT)/computed tomography (CT) for up to 40 days, and the results were validated *ex vivo*. Using this approach, we demonstrated that Treg trafficking to skin grafts was regulated by the presence of recipient Gr-1$^+$ innate immune cells. We demonstrated the utility of radionuclide reporter gene-afforded quantitative Treg *in vivo* tracking, addressing a fundamental need in Treg therapy development and offering a clinically compatible methodology for future Treg therapy imaging in humans.

INTRODUCTION

Regulatory T cells (Tregs) are immunosuppressive cells with a fundamental role in maintenance of tolerance to self-antigen *in vivo*.[1,2] Tregs are characterized by constitutively high expression of CD25 and the master transcription factor forkhead-box protein 3 (FOXP3). Mice deficient in Foxp3 exhibit systemic autoimmune disease, and their Tregs have an impaired suppressive capacity *in vitro*.

In transplantation, graft rejection is a detrimental process driven by alloreactive T cells that recognize donor major histocompatibility complex (MHC) antigens (alloantigens) via various pathways.[3] In addition to maintaining tolerance to self-antigens, Tregs contribute to controlling responses to alloantigens, and a correlation between the proportion of Tregs within a transplanted organ (allograft) and graft survival has been observed.[4–6] This has led to the pursuit of protocols designed to tip the balance between Tregs and effector T cells (Teffs) in favor of Tregs to induce transplantation tolerance. One strategy to increase Treg numbers is purification of Tregs from allograft recipients, expanding and potentially manipulating them *in vitro* before re-administration. In immunodeficient mice transplanted with human skin and reconstituted with peripheral blood mononuclear cells (PBMCs), adoptive transfer of human *in-vitro*-expanded polyclonal Tregs significantly prolonged the survival of skin allografts.[7,8] Polyclonal Treg-based cell therapy approaches yielded early promising results for prevention of graft versus host disease (GvHD), after allogeneic hematopoietic stem cell transplantation[9,10], and for maintenance of C-peptide levels in type I diabetes.[11,12] We led two clinical phase I/II trials using adoptive transfer of polyclonal expanded Tregs to promote tolerance in kidney (ONE Study, NCT02129881) and liver (ThRIL, NCT02166177) transplant recipients.[13–15] The resultant data

Received 7 September 2020; accepted 2 December 2020;
https://doi.org/10.1016/j.omtm.2020.12.003.

[6]Present address: Molecular Imaging Group, Department of Radiology, Memorial Sloan Kettering Cancer Center, 417 E 68th St., New York, NY 10065, USA

[7]Senior author

Correspondence: Giovanna Lombardi, School of Immunology and Microbial Science, King's College London, Guy's Hospital, Tower Wing, 5th Floor, Great Maze Pond, London SE1 9RT, UK.
E-mail: giovanna.lombardi@kcl.ac.uk
Correspondence: Gilbert O. Fruhwirth, School of Biomedical Engineering and Imaging Sciences, King's College London, St. Thomas' Hospital, Lambeth Wing, 4th floor, Westminster Bridge Rd., London SE1 7EH, UK.
E-mail: gilbert.fruhwirth@kcl.ac.uk

 © 2021 The Authors.
This is an open access article under the CC BY license (http://creativecommons.org/licenses/by/4.0/).

suggested Treg therapy to be safe and well tolerated and showed some signs of efficacy.

Despite an increase in the numbers of clinical trials using human Treg therapy, important questions pertaining to their *in vivo* fate, distribution, and location of their function remain unclear. Recently, polyclonal Tregs labeled with [6,6-$^{2}H_2$]glucose were detected in the circulation of individuals with type I diabetes for up to one year.[12] However, it remains a challenge to identify adoptively transferred Tregs in tissues non-invasively.

Non-invasive radionuclide imaging by single-photon emission computed tomography (SPECT) or positron emission tomography (PET) offers excellent sensitivity with absolute quantification and true 3D information while being translatable to the clinic. Cell labeling approaches can be direct or indirect, with each approach offering distinct advantages and disadvantages.[16] We showed previously that direct radiolabeling of murine CD4^{+} T cells, with ^{99m}Tc-hexamethylpropyleneamine oxime, did not affect cell viability, but the radiolabeled cells could only be tracked for up to 24 h because of the short radioisotope half-life of ^{99m}Tc (6 h).[17] This enabled assessment of Treg biodistribution within a day after administration but precluded the desired long-term tracking of Tregs, which would require longer half-life radioisotopes. Longer half-life radioisotopes are available (e.g. ^{111}In and ^{89}Zr) but also elicit a higher radioactive dose,[18] limiting the study length. Using various T lymphocytes, recent studies involved tracking of about one week but not longer.[19–21] In the context of transplant immunology, aiming to track Tregs to transplants, one week is a short observation time, and significantly longer cell tracking would be beneficial.

Long-term cell tracking can be achieved through indirect cell labeling, which involves genetic engineering to express a reporter and render the cells *in vivo* traceable by repeat imaging.[16,22] Treg *in vivo* dynamics were assessed using bioluminescence approaches.[23–25] However, bioluminescence imaging is not translatable to the clinic because of the non-human nature of luciferases, which also require luminogenic substrate administration, and the added disadvantages of optical imaging at depth (absorption, scatter) precluding reliable quantification. Radionuclide reporter genes are an alternative that enables 3D tomographic imaging, and they have also been shown to be clinically translatable.[26] Host reporter genes are from the same species as cell tracking should occur in and should be endogenously expressed in only a very limited number of host tissues to ensure favorable contrast and overcome any immunogenicity issues intrinsic to foreign reporters.[16,22] One of the most promising host reporter genes is the human sodium iodide symporter (NIS), which has been shown to be well tolerated in different cell types, including cancer cells, cardiomyocytes, stem cells, and T cells.[27–30] In a proof-of-principle study employing retroviral transduction methodology, we demonstrated *ex vivo* engineering of murine Tregs to express NIS and detected the cells *in vivo* 24 h after administration by SPECT imaging.[31] Importantly, it has also been shown recently that the radioactivity levels that can be expected to be taken up into T cells through NIS use during imaging did not cause lasting DNA damage in T cells.[32] Moreover, the matching radiotracers required for NIS imaging have already been translated to humans (for thyroid imaging).[33,34] These features make NIS a well-established reporter gene suitable for preclinical long-term cell tracking and a candidate reporter for the clinical setting. Notably, so far, long-term *in vivo* tracking of human Tregs has not been addressed using a reporter gene imaging approach that can be translated to the clinic.

Our goal was to genetically engineer GMP-isolated and *in-vitro*-expanded human Tregs, characterize the resultant traceable Tregs, and apply them to investigate their capacity to home to and reside within human skin transplants in a humanized mouse model. Because it has been reported that, during inflammation, soluble mediators (for example, chemokines produced by infiltrating granulocytes and monocytes) are critical for recruitment of adaptive immune cells to antigen-rich tissues,[35] we applied our approach to investigate whether innate immune cells had an effect on Treg trafficking to skin grafts.

RESULTS

Generation of *in-vivo*-traceable Tregs

CD4^{+}CD25^{high} Tregs from donor blood were enriched using GMP protocols[13–15] and stimulated with anti-CD3/CD28 beads in the presence of rapamycin and interleukin-2 (IL-2) (Figure S1). Three days after stimulation, Tregs were transduced with lentiviral particles transferring DNA encoding the radionuclide-fluorescence fusion reporter gene NIS-GFP (Figure 1A; monomeric GFP aides traceable Treg generation, purification, and *ex vivo* detection[27,28,36]). Transduced NIS-GFP^{+} Tregs were purified by cell sorting (purity > 99%; Figure 1B), and NIS-GFP was found predominantly in the plasma membrane, suggesting correct intracellular reporter trafficking (Figure 1C). No significant changes in reporter expression levels were observed over six weeks, suggesting stable NIS-GFP expression (Figure 1D). Importantly, we also did not find differences in Treg expansion between untransduced and reporter-expressing Tregs (Figure 1E). NIS-GFP^{+} Tregs showed uptake of the radioactive NIS substrate [^{99m}TcO$_4^{-}$] whereas untransduced Tregs did not (Figure 1F). [^{99m}Tc]TcO$_4^{-}$ uptake was also reduced significantly in NIS-GFP^{+} Tregs in the presence of excess amounts of the NIS co-substrate perchlorate, demonstrating radiotracer uptake to be specific for NIS-GFP expression (Figure 1F).

NIS-GFP^{+} Tregs maintain phenotype and function

To investigate whether reporter transduction and radiolabeling affect the Treg phenotype, we compared NIS-GFP^{+} Tregs with untransduced Tregs employing established Treg markers (CD25, FOXP3, and CTLA-4). This comparison was performed with identical Treg batches that had been exposed to [^{99m}Tc]TcO$_4^{-}$ radiotracer or vehicle, and no significant effect of NIS transduction and NIS use (radiolabeling) was detected (Figures 2A and 2B; Figure S2). Next we assessed the suppressive capacity of NIS-GFP^{+} Tregs

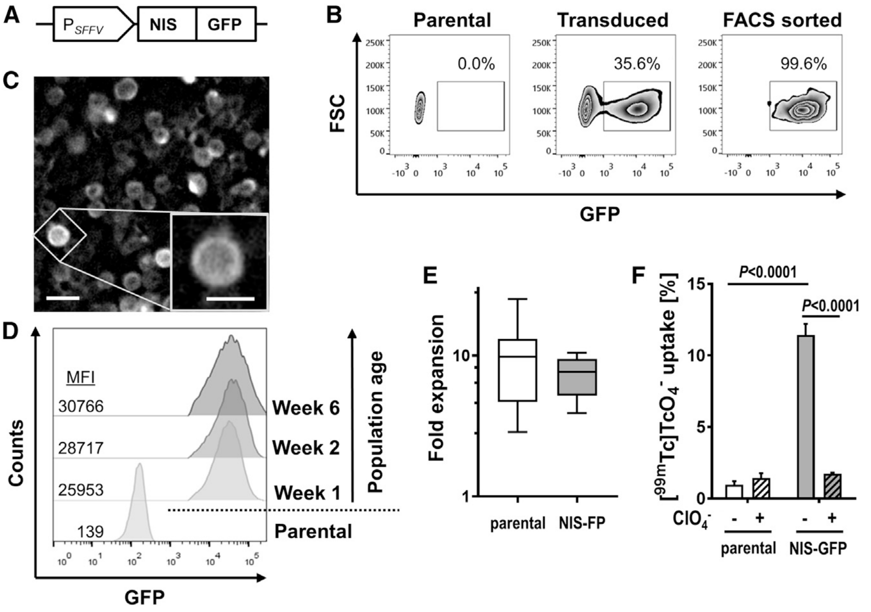

Figure 1. Generation of Tregs stably expressing NIS-GFP

(A) Sketch of the lentiviral construct. SFFV, spleen focus-forming virus promoter. (B) Representative example of Treg transduction with the NIS-GFP reporter. Post-transduction FACS yielded more than 99% pure NIS-GFP$^+$ Tregs. (C) Fluorescence microscopy revealed NIS-GFP expression in Tregs to be predominantly membranous. Scale bars, 10 μm and 5 μm (inset). (D) Flow cytometry analysis of NIS-GFP$^+$ Tregs in the weeks after transduction alongside corresponding untransduced Tregs (numbers indicate mean fluorescence intensity [MFI]). One representative of three experiments is shown. (E) Transduced NIS-FP$^+$ Tregs were sorted by FACS (*cf.* B) and expanded further for subsequent experiments over a period of ten days. Fold expansion of these cells during this period was quantified and compared with corresponding untransduced parental Tregs. Shown is a box-and-whisker plot with median and 25th–75th percentiles indicated as well as min and max values drawn as whiskers; n = 9. There was no significant difference between the cell types. (F) NIS-GFP function as quantified per [^{99m}Tc]TcO_4^- uptake in untransduced and NIS-GFP$^+$ Tregs (gray). $NaClO_4$ block reports NIS specificity (black). n = 3, error bars indicate SD; ANOVA with Tukey multiple comparisons correction.

compared with untransduced Tregs based on their ability to suppress the proliferation of dye-labeled Teffs. No significant differences between NIS-GFP$^+$ Tregs and untransduced Tregs were found, neither in the presence nor in the absence of exposure to the NIS radiotracer [^{99m}Tc]TcO_4^- at different Teff:Treg ratios (Figure 2C; Figure S3). Importantly, these data demonstrated that (1) NIS-GFP$^+$ Tregs maintained their suppressive function after lentiviral transduction and upon stable reporter expression, and (2) the amount of [^{99m}Tc]TcO_4^- used, which is comparable to what NIS-GFP$^+$ Tregs would experience upon reporter gene imaging *in vivo*, did also not impair their function.

These data showed that human Tregs genetically engineered via lentiviral transduction technology to stably express the radionuclide-fluorescence reporter NIS-GFP maintained their phenotype and suppressive function following labeling.

In vivo detection of NIS-GFP$^+$ Tregs in a humanized mouse model

First we assessed the detection sensitivity of [^{99m}Tc]TcO_4^--labeled NIS-GFP$^+$ Tregs in our nanoSPECT/computed tomography (CT) instrument and found a limit of detection (LOD) of ~10,000 NIS-GFP$^+$ Tregs per million untransduced cells (Figure S4). These data demonstrated the feasibility of Treg tracking by NIS reporter gene imaging.

Next we determined the traceability of NIS-GFP$^+$ Tregs *in vivo* in a well-established human skin xenograft transplant model. Previously, we had demonstrated the efficacy of human polyclonal Tregs to reduce alloimmune human skin injury (30 days after administration).[7,12] Therefore, human skin grafts were prepared using immunocompromised BALB/c recombination activating gene $2^{-/-}\gamma c^{-/-}$ (BRG) mice as recipients. Six to seven weeks after skin engraftment, transplant-bearing mice were randomized, and allogeneic CD25-depleted PBMCs were administered intravenously alone or together with NIS-GFP$^+$ Tregs. Importantly, murine granulocyte receptor 1 (Gr-1)-positive cells, including neutrophils and certain monocytes, were still functional in BRG mice. To achieve a higher level of immunocompromise, Gr-1$^+$ cells were depleted using an antibody raised against Gr-1 as described previously (Figure 3A).[7,12,38] It is noteworthy that despite this being an established approach to deplete the remaining Gr-1$^+$ cell using the indicated antibody, we confirmed its success by histology (Figure S5). Treg *in vivo* detection was based on visualization of NIS-GFP$^+$ Tregs by SPECT imaging with the NIS radiotracer [^{99m}Tc]TcO_4^-. Notably, the host reporter gene human NIS is homologous to murine NIS (mNIS), and the radiotracer detected both forms.[37] This explained why we observed, in mice that did not receive NIS-GFP$^+$ Tregs (Figure 3C), the following signals attributed to endogenously expressed mNIS: thyroid, salivary, and lachrymal glands; the stomach; and, at lower levels, the small intestine and the testes of male mice. Bladder signals were caused by the expected renal radiotracer excretion. Importantly, in animals that had received NIS-GFP$^+$ Tregs, we detected the traceable therapeutic cells in the transplants (Figure 3D). Importantly, none of the aforementioned organs with endogenous mNIS expression or involved in renal radiotracer excretion contributed to any background signals in human skin grafts (cf. Figures 3C and 3D, insets), enabling quantification of NIS-GFP$^+$ Tregs in the skin grafts.

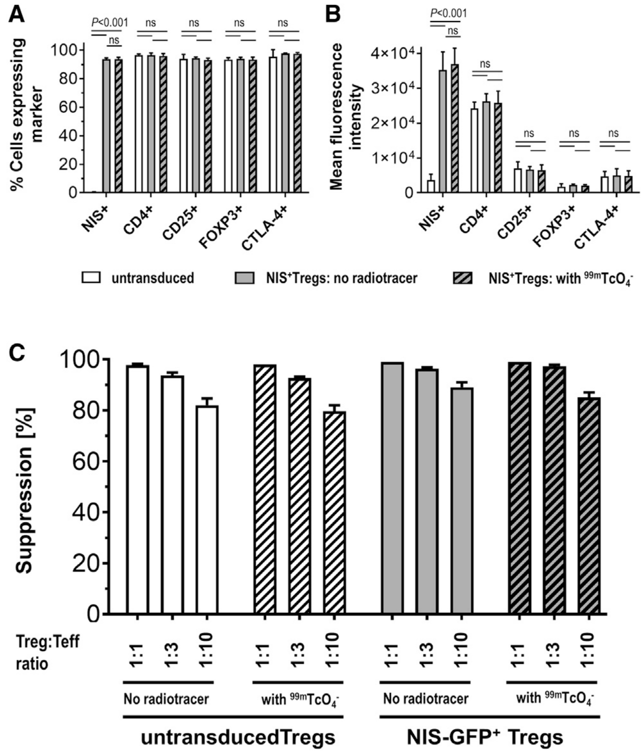

Figure 2. Assessment of marker expression and suppressive function

(A and B) The Treg markers CD25, FOXP3, and CTLA-4 were quantified by flow cytometry alongside CD4 (all via antibody staining) and NIS-GFP (via intrinsic GFP fluorescence). NIS-GFP$^+$ Tregs were analyzed by flow cytometry before and six days after [^{99m}Tc]TcO_4^- uptake. Cumulative data describe (A) the percentage and (B) the mean fluorescence intensities. n = 3, error bars indicate SD, Student's *t*-test with Holm-Sidak multiple comparisons correction for both (A) and (B). For representative individual data, see Figure S3. (C) Comparison of suppressive capacity of untransduced and NIS-GFP$^+$ Tregs with or without radiotracer treatment. Shown are cumulative data of three different Treg batches (donors); error bars indicate SD. There were no significant differences between cells with or without radiotracer treatment (Student's *t*-test; untransduced Tregs: $p_{1:1}$ = 0.3739; $p_{1:3}$ = 0.2508; $p_{1:10}$ = 0.3139; NIS-GFP$^+$ Tregs: $p_{1:1}$ = 0.3739; $p_{1:3}$ = 0.1012; $p_{1:10}$ = 0.1194). For representative individual data, see Figure S3.

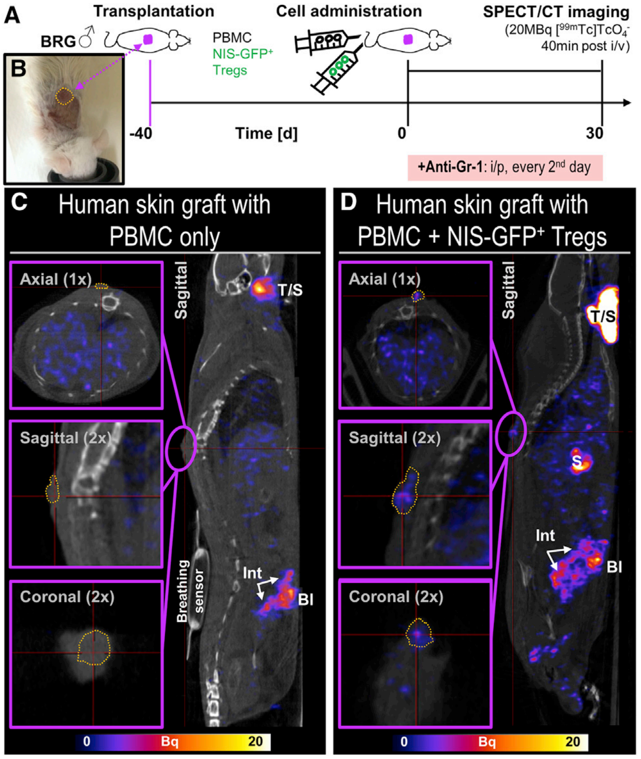

Figure 3. *In vivo* detection of NIS-GFP$^+$ Tregs in human skin grafts

(A) Experimental scheme, including imaging conditions (radiotracer and time between its administration and SPECT imaging). (B) Representative example of a human skin transplant atop the spine of a BRG mouse on the day of adoptive immune cell transfer. All animals in this experiment (n = 3 in each group) received the same human skin and Tregs from the same donor; all mice were depleted of Gr-1$^+$ innate immune cells. (C and D) Sagittal sections of SPECT/CT images from representative animals that received 5 × 10^6 CD25-depleted PBMCs but (C) no NIS-GFP$^+$ Tregs or (D) 5 × 10^6 NIS-GFP$^+$ Tregs 30 days prior to imaging. Overlays of SPECT images in hue (and on the same scale) with co-registered CT images (grayscale) are shown. Purple frames indicate magnified areas in different planes, with the human skin graft circumscribed by dark yellow dotted lines; thin red crosshair lines indicate how the indicated sections connect to one another. Endogenous (murine) mNIS expressing organs are visible in both groups and include thyroid/salivary glands (T/S), stomach (S), and small intestine (Int),[37] whereas bladder (Bl) signals are caused by radiotracer excretion. Images demonstrate (C) that the transplant area atop the spine remained free of any SPECT signals when no NIS-GFP$^+$ Tregs were administered and (D) that administered NIS-GFP$^+$ Tregs were detected by SPECT imaging at the skin graft as they trafficked to this site over time.

Serial *in vivo* imaging revealed a role of Gr-1$^+$ innate immune cells in Treg recruitment

Next we used serial SPECT/CT imaging to track NIS-GFP$^+$ Tregs at different time points after intravenous administration into transplant-bearing immunocompromised mice lacking Gr-1$^+$ innate immune cells. BRG mice received traceable NIS-GFP$^+$ Tregs and were treated with the anti-Gr-1 antibody to deplete this cell compartment (Figure S5). We found that radiotracer uptake levels in their human skin grafts did not significantly differ from control animals (no NIS-GFP$^+$ Tregs and only CD25-depleted PBMCs administered) for the first 2 weeks (cf. pink versus black lines in Figure 4). Notably, NIS-GFP$^+$ Treg presence was elevated significantly compared with control animals at late time points, ranging 30–40 days after administration (Figure 4). Using non-obese diabetic (NOD).Cg-Prkdc^{scid} Il2rg^{tm1Wjl}/SzJ (NSG) mice, which have less and mostly dysfunctional innate immune cells[39] and thus did not receive the anti-Gr-1 antibody, we obtained similar results compared with those obtained in BRG mice with Gr-1$^+$ cell depletion (Figure S6).

Next, to investigate the role of Gr-1$^+$ innate cells on Treg recruitment to skin grafts, we determined the *in vivo* trafficking behavior of intravenously (i.v.) administered NIS-GFP$^+$ Tregs in transplant-bearing BRG mice without anti-Gr-1 antibody administration (Figure 4; Figure S5). Interestingly, NIS-GFP$^+$ Tregs were detectable at the skin

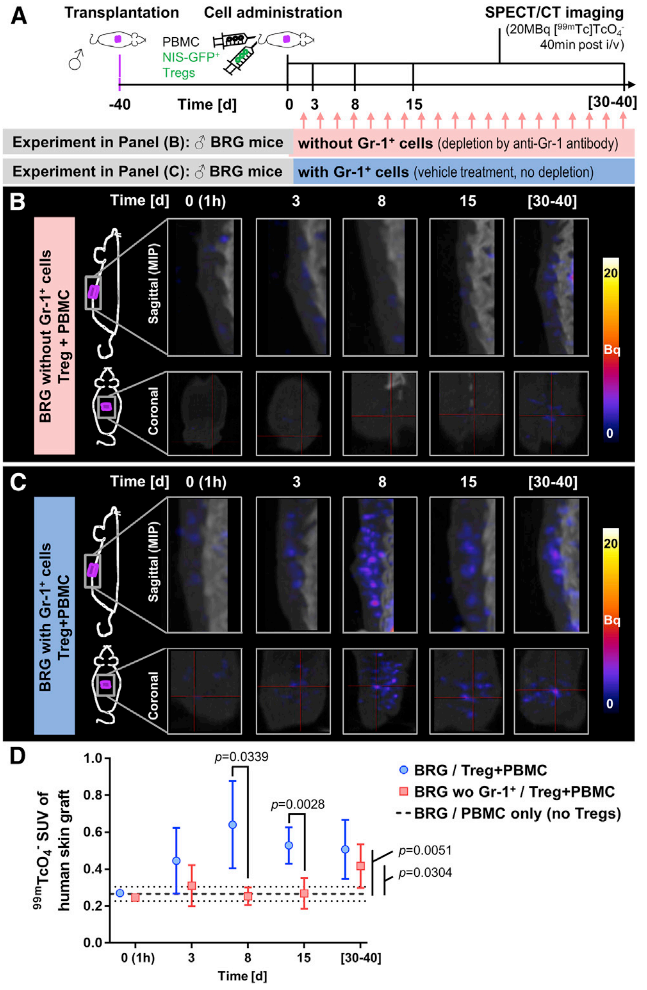

Figure 4. *In vivo* tracking of NIS-GFP⁺ Treg arrival at human skin grafts

(A) Experimental scheme. Purple, human skin graft; green syringe, 5 × 10^6 NIS-GFP⁺ Tregs; black syringe, 5 × 10^6 PBMCs. Cohorts were randomized upon adoptive cell transfer. (B and C) Longitudinal SPECT/CT images of transplant-bearing animals at the indicated time points after adoptive cell transfer. Images were acquired 40 min after $[^{99m}Tc]TcO_4^-$ administration (20 MBq). Side-view maximum intensity projections (top row) and coronal sections through the graft (bottom row) are shown; all images are on the same scale. Radioactive signals above background in grafts indicate NIS-GFP⁺ Treg presence. Shown are representative images from the same animal. (D) Cumulative quantitative analysis of *in vivo* imaging data. SUV, standard uptake value. Black dashed/dotted lines represent mean ± SD of pooled control animals (PBMCs/no Tregs and no adoptive transfer; n = 5). Shown are cumulative data of all experiments in (B) and (C); n = (7;7;7;5 for (B)) and (6;4;5;4 for (C)) for the time points day (3;8;15; range [30–40]), respectively. Error bars represent SD. Multiple *t*-tests with Holm-Sidak correction for multiple comparisons were used for statistical analysis and p-values are shown if < 0.05.

grafts as early as 3 days after administration. Their signals peaked at around 8 days and remained detectable in the transplants up to 40 days after administration (Figure 4C). Image quantification revealed significant differences between BRG mice without and with $Gr\text{-}1^+$ cell depletion at early time points after Treg administration (Figure 4D). Early trafficking of Tregs to the skin in the presence of $Gr\text{-}1^+$ cells suggested an active role of these cells in influencing Treg recruitment to the graft.

Ex vivo validation of *in vivo* imaging

Animals of each cohort were sacrificed at the indicated time points, and radioactivity in the graft tissues was quantified (by γ- counting). Human skin grafts were compared with mouse skin from the same animal using paired analyses. In control animals that did not receive traceable Tregs, no significant differences in tissue radioactivity between human grafts and mouse skins at any time point were observed (Figure 5A, solid versus dashed black lines), demonstrating no human graft-specific radiotracer accumulation. In BRG mice that had also received anti-Gr-1 antibody treatment, we observed no significant differences between human and mouse tissues for the first 2 weeks but identified a significant difference between the two skins types on day 40 ($p = 0.0309$; Figure 5A, compare solid versus dashed pink lines). Importantly, in BRG mice with $Gr\text{-}1^+$ cells present, the radioactivity found in human skin grafts was higher than in mouse skin at all time points ($p = 0.0016$; Figure 5A, solid versus dashed blue lines). These comparisons of human skin grafts with mouse skin from the same animals clearly demonstrated a preference for traceable human Tregs to accumulate at human skin grafts.

Human skin grafts from different animal cohorts were compared using unpaired statistical analysis. The radioactivity levels in human skin grafts on day 8 were significantly higher in BRG mice with $Gr\text{-}1^+$ cells present compared with BRG mice lacking $Gr\text{-}1^+$ cells and control cohorts (Figure 5A). Notably, there were no differences between mouse skins of the same three animal cohorts. On day 40, the situation had changed, with both experimental cohorts (BRG with $Gr\text{-}1^+$ cells and BRG without $Gr\text{-}1^+$ cells) showing significantly elevated radioactivity in human skin grafts compared with control animals ($p = 0.0340$ and $p = 0.0071$, respectively; Figure 5A; ANOVA with Holm-Sidak multiple comparison correction). These data further confirmed our *in vivo* imaging data.

Moreover, we used immunofluorescence histology to independently validate the NIS-GFP⁺ Treg presence in human skin grafts. First, in control animals that had received only CD25-depleted human PBMCs, we found human $CD3^+$ (Figure 5B), whereas in additional control mice that had only received saline, we did not observe the presence of any human $CD3^+$ cells (Figure 5C). In BRG mice that were treated with the anti-Gr-1 antibody, we detected human $CD3^+FOXP3^+$ Tregs alongside human $CD3^+$ in the skin grafts on day 40 (Figure 5D). Importantly, we also found human $CD3^+FOXP3^+$ Tregs in the human skin grafts of BRG mice with $Gr\text{-}1^+$ cells present on day 8 and day 40 (Figures 5E and 5F), again confirming our previous results. We also obtained *ex vivo* results in line with *in vivo* imaging data for NSG mice (Figures S5D and S5E).

In summary, our *in vivo* imaging data were confirmed by two independent *ex vivo* tissue analyses and demonstrated murine $Gr\text{-}1^+$ cells to affect human Treg arrival in human skin graft models.

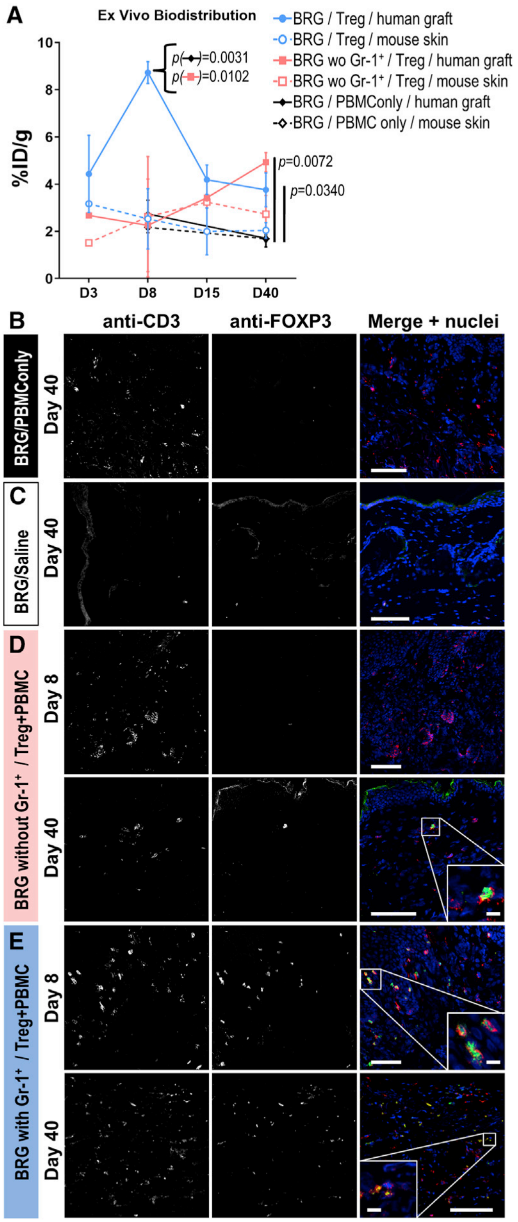

Figure 5. *Ex vivo* tissue analysis of transplanted tissues

(A) *Ex vivo* radioactivity analysis of harvested tissues. Mouse skin was harvested from a region above the lower spine. Human skin grafts were compared with mouse skin from the same animal using paired *t*-tests. No Treg/PBMC-only control: not significant over all time points ($p = 0.2526$), solid versus dashed black lines; BRG without Gr-1$^+$ cells: not significant for early time points ($p = 0.4850$) but significant on day 40 ($p = 0.0309$), solid versus dashed pink lines; BRG with Gr-1$^+$ cells present: significant over all time points ($p = 0.0016$), solid versus dashed blue lines. Human skin grafts from different animal cohorts were compared using unpaired ANOVA on day 8 or day 40, with p values shown in (A). Cumulative data; n = 2 (days 3 and 15) and n = 4 (days 8 and 40); error bars indicate SEM. (B–E) Immunofluorescence staining of harvested tissues with anti-human CD3 (red in merged images) and anti-FOXP3 antibodies (green in merged images). Indicated times refer to days after adoptive Treg transfer as in Figure 4. Shown are (B) untreated mice that received only PBMCs or (C) no cells (saline only). (D) Representative confocal micrographs of Gr-1$^+$ cell-depleted mice, which received PBMCs and NIS-GFP$^+$ Tregs. (E) experiments as in (C) but performed in BRG mice without Gr-1$^+$ cell depletion. CD3 staining is indicative of administered PBMC-derived human T cells and Tregs, whereas FOXP3 staining identified administered human Tregs only. Scale bars are 100 μm.

Human Treg migration is enhanced by murine neutrophils/monocytes *in vitro*

To gain more mechanistic insights into the role of innate immune cells on Treg recruitment, we tested whether murine neutrophils and/or monocytes could affect human Treg migration using *in vitro* transmigration assays.

First we analyzed whether human Tregs responded to the murine chemokines CCL17/CCL22. It has been reported previously that Tregs are responsive to gradients of chemokines engaging CCR4,[40,41] and we confirmed here that human Tregs expanded *in vitro* according to our GMP protocol continued to express high levels of CCR4 (Figure S7). The same human Treg preparations used for *in vivo* tracking experiments (see Figure S8A for individual donors) were seeded into the top well of a Transwell system, and their migration through a 3 μm porous membrane was quantified, with the bottom chamber containing medium with the indicated admixtures. Under some conditions, Tregs were pre-treated with pertussis toxin (PTX). The murine chemokines CCL17/CCL22 were able to increase human Treg migration ($p < 0.0001$, two-way ANOVA), an effect that was reversed by PTX pre-treatment of Tregs (Figures 6A and 6B), suggesting G-protein-coupled receptor (GPCR) involvement.

Having established that human Tregs responded to murine pro-migratory stimuli, we then investigated whether isolated murine neutrophils and/or monocytes (Figures S8B and S8C) could induce human Treg migration. To exclude any direct cell-cell interactions, neutrophils/monocytes were provided in the bottom chambers, whereas human Tregs were added to the top well of the Transwell system. Notably, neutrophils and monocytes were activated in this experiment because of the use of zymosan during their preparation (Materials and methods). Neutrophils and monocytes as well as their combination significantly increased human Treg migration (Figures 6C and 6D; $p = 0.0112$, two-way ANOVA). A synergistic effect of neutrophils and monocytes was not observed. Notably, enhancement of human Treg motility was reduced when they were pre-treated with PTX, with no significant migration differences detected between the presence or absence of the indicated cell types (Figures 6E and 6F; $p = 0.2478$, two-way ANOVA).

Taken together, murine Gr-1$^+$ cells accelerated human Treg recruitment to inflammatory sites (such as human skin graft models) by production of GPCR ligands, likely chemokines, which cross-reacted with GPCRs on human Tregs.

DISCUSSION

Previously, we have demonstrated the efficacy of human polyclonal Tregs at reducing alloimmune injury in a human skin xenograft

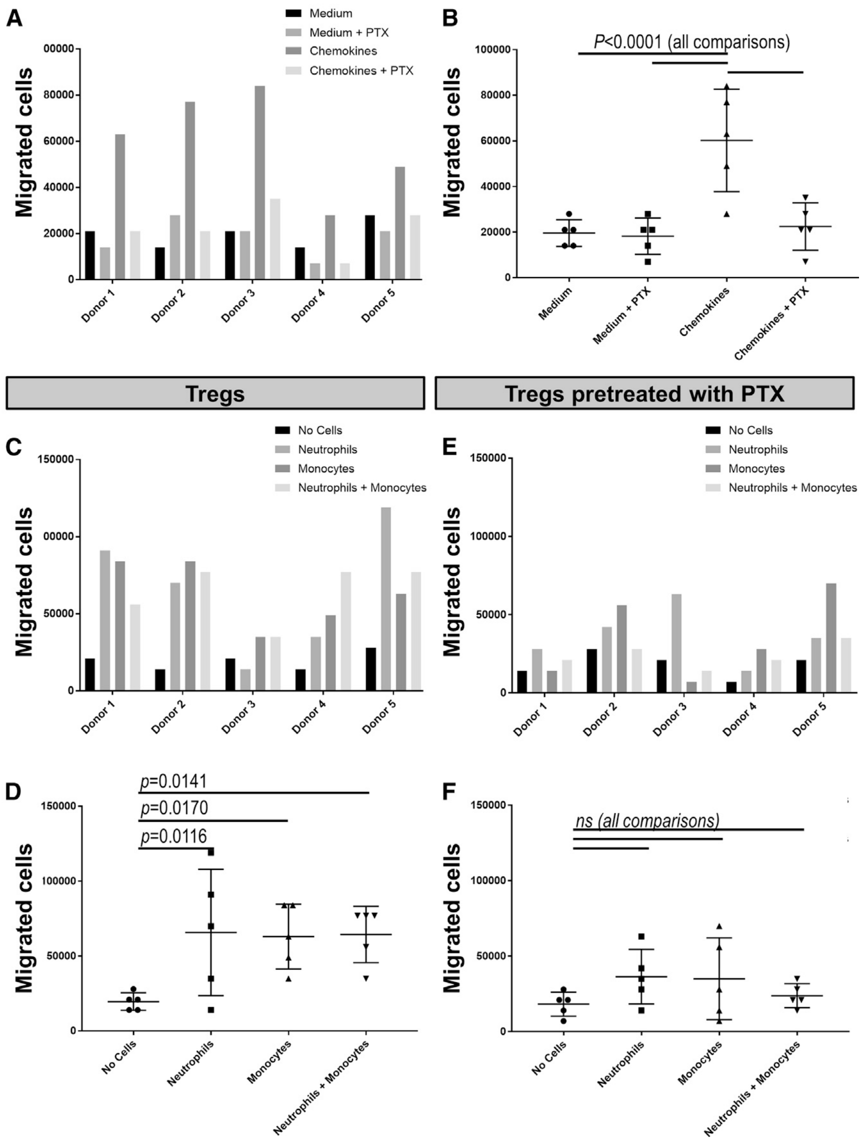

Figure 6. *In vitro* Treg migration assay

(A) Transwell migration assay (3 μm pore size) of human Tregs from five different donors (top chamber). The bottom chambers contained growth medium without or with the chemokines CCL17/CCL22. PTX indicates that Tregs were pre-treated with pertussis toxin (PTX). (B) Cumulative data of (A) showing mean ± SD. Two-way ANOVA revealed significant differences ($p < 0.0001$). Migration increased significantly with CCL17/CCL22 ($p = 0.0001$ with Dunnett's multiple comparisons test) compared with other conditions. (C) Transwell migration assay as in (A) but with the bottom chambers containing the following purified murine cells: none, neutrophils, monocytes, and neutrophils and monocytes. Data are grouped per donor. (D) Cumulative data of (C) showing mean ± SD. Two-way ANOVA revealed significant differences with different cells admixed ($p = 0.0112$). Dunnett's multiple comparisons test revealed significant differences for each cell admixture compared with medium only (see p values in the figure). (E and F) Data from the same experiments as in (C) and (D), but Tregs were pre-treated with PTX. No significant differences were found between the groups by the same statistical tests as in (C) and (D).

transplant model.[7,8] However, little was known about the *in vivo* distribution and fate of administered Tregs, and it remained extremely challenging to track adoptively transferred Tregs non-invasively in solid tissues. We previously attempted to assess the *in vivo* distribution of murine Tregs by whole-body imaging within a day of administration.[17,31] What remained elusive was long-term tracking as well as tracking of human Tregs in skin transplant models to quantify their arrival at transplant sites.

Therefore, we used the well-established dual-mode reporter NIS-GFP[28] to enable long-term *in vivo* tracking. The Achilles heel of all reporter gene-afforded cell tracking is the additional regulatory hurdle upon clinical translation because of the need to genetically engineer the cell product. However, chimeric antigen receptor (CAR) Treg therapy has recently emerged as the next-generation strategy for Treg therapy. We and others have already successfully generated and characterized prototype CAR Tregs for various indications, including colitis and multiple sclerosis and in humanized mouse models of xeno-GvHD and human skin transplantation.[38,42–44] We performed gene transfer into Tregs using lentiviral technology, which has been used successfully for genetic engineering of cell-based therapeutic agents in the clinic before (e.g., anti-cancer CAR T cell therapeutic agents).[45,46] The first CAR Treg clinical trial has been granted by UK Medicines and Healthcare products Regulatory Agency (MHRA) authorization: a phase I/II clinical trial (STEADFAST) for kidney transplant patients. Therefore, investigation of the suitability of reporter gene technology for therapeutic Tregs was worthwhile because it provided another mosaic piece in the journey to further develop and translate Treg/CAR Treg therapy for human use.

The most promising host reporters available for our purpose were NIS[37] and prostate-specific membrane antigen[47] (for a review of host reporter genes for immune cell tracking, see Ashmore-Harris et al.[16]), neither of which was expressed in human or mouse dermis or epidermis. NIS offered the local technical advantage of a generator-produced radiotracer ([^{99m}Tc]TcO_4^-) for SPECT imaging, avoiding radiotracer synthesis on each imaging day. Preclinically, the sensitivity advantages of PET over SPECT are not so pronounced on high-quality instrumentation; however, clinically, PET is superior

to SPECT in sensitivity. Importantly, a clinical PET tracer for NIS is already available ([^{18}F]BF_4^-)[28,33] and will be particularly useful in the future when combined with new total-body PET instrumentation that has been shown to offer further significant sensitivity improvements (i.e., 40-fold improvement of total-body PET over existing clinical PET[48]). Moreover, NIS has been reported to be very sensitive to cell viability because of its dependence on the cellular Na^+/K^+ gradient,[37] enabling reliable imaging of live cells only. The fluorescent co-reporter GFP in our fusion reporter NIS-GFP helped streamline generation of traceable cells for preclinical experimentation.[27,28,36] Notably, NIS-fluorescent protein fusion reporters have been shown previously (1) to be correctly localized to the plasma membrane and function in several different cell types (rodent and human cancer cells,[20,27–29,36] human CAR T cells,[49] and hepatocyte-like cells[29]) and (2) to mediate uptake of various substrates similarly to unmodified NIS (for similar relative substrate uptake of NIS substrates, compare Figure S9A, Diocou et al.[36], and Khoshnevisan et al.[50] with Portulano et al.[37] and Dohán et al.;[51] for comparative considerations relating to the NIS Michaelis-Menten constant for tetrahedral substrates, see Figure S9B). Importantly, although NIS-GFP was practical for preclinical experimentation, removal of GFP will be required to render our Treg tracking approach fully compatible with the workflows required for human application. Consequently, selection conditions will need to change, but suitable NIS antibodies or alternative selective expansion strategies[52] are already available.

One apparent shortcoming of radionuclide reporter gene imaging approaches is their limited spatial resolution (low millimeter range). This can be overcome by combining them with technologies that provide routinely about an order of magnitude higher resolution and anatomical context, such as X-ray CT or magnetic resonance imaging (MRI).[53] Because this generally leads to better interpretability of obtained co-registered images by combining the best of both worlds, we also adopted a multi-modal SPECT-CT imaging approach throughout this work.

In this work, we generated Tregs stably expressing NIS-GFP for a minimum of 6 weeks (Figure 1); we did not detect any negative effect of lentiviral transduction on Treg expansion (Figure 1) or on Treg phenotypes or suppressive function (Figure 2). Importantly, there was also no negative effect of radiolabeling NIS-GFP$^+$ Tregs with the SPECT radiotracer [^{99m}Tc]TcO_4^- followed by full radioactivity decay (Figure 2), lending credibility to the overall reporter imaging strategy to be safe for long-term monitoring of Tregs (in line with previous observations that it is safe in Teffs[32]). We found the LOD for NIS-GFP$^+$ Tregs in our instrument to be $\sim 10^4$ cells/million (Figure S4), roughly one order of magnitude lower than what we found previously for cancer cells;[27] we attributed this to the Tregs being about ~2-fold smaller in diameter (amounting to ~$^1/_8$ in volume, taking up at least 8-fold less radiotracer per cell). In this setting, we were able to track NIS-GFP$^+$ Tregs for 40 days after administration, only limited in time by animal license regulations, and *ex vivo* data confirmed *in vivo* results (Figures 4 and 5). Future work will need to focus on more in-depth analysis of potential reporter gene effects and put emphasis on potential transcriptomic, proteomic, and metabolomic changes.

Previous work in mice (for example, in an islet allograft model) demonstrated that Tregs migrated from the blood stream first to inflamed allografts and subsequently to draining lymph nodes.[54,55] The latter phenomenon was not detected in our studies, not because of limited detection sensitivity but because of the lack of functional lymph nodes in the immunocompromised mice used in this humanized model. Several studies in cancer and pregnancy research have suggested roles of neutrophils or monocyte-derived suppressor cells (MDSCs) in induction and recruitment of Tregs to tumors.[56–58] Notably, MDSCs were first identified to play an important role in tolerance induction in a rat kidney transplant model.[59] There are two MDSC subtypes, monocytic (M)-MDSCs and polymorphonuclear/granulocytic (PMN/G)-MDSCs, which show some phenotypic similarity to monocytes or neutrophils, respectively, and both present with immune-suppressive functions. Furthermore, adoptive transfer experiments with M-MDSCs and PMN-MDSCs in a heart transplant model demonstrated a role of these MDSC in Treg expansion in the transplant and for prolonged allograft survival.[60] Despite much research, not least in human transplantation, where a positive correlation between MDSCs and Tregs has been observed[61,62], the detailed mechanisms by which MDSCs induce Tregs still remain unclear. Treg migration to inflamed islet allografts, however, has been found to depend on CCR2, CCR5, and, importantly, CCR4.[54] Neutrophils and MDSCs produce the chemokines CCL17/CCL22, which are ligands for CCR4 (Iellem et al.;[40] Figure S7), and expression of this chemokine receptor by Tregs has been implicated previously in their migration to allografts.[54] Furthermore, CCL17 has also been shown to be involved in neutrophil-mediated Treg recruitment to tumors.[58] Gr-1$^+$ murine innate immune cells comprise neutrophils and subsets of monocytes and dendritic cells. A Gr-1$^+$ monocyte subset has been observed previously in a cardiac transplant model,[63] and Gr-1$^+$ monocytes have also been shown to differentiate into tolerogenic dendritic cells that produced IL-10 and induced Treg responses *in vivo* in tumors.[64] We hypothesized that murine Gr-1$^+$ cells might also affect Treg recruitment in human skin transplant models. *In vivo* tracking of NIS-GFP$^+$ Tregs in human skin transplant-bearing BRG mice that were either left untreated or depleted of Gr-1$^+$ murine cells revealed significant differences in NIS-GFP$^+$ Treg recruitment to the grafts (Figures 4 and 5). These results were supported by data from NSG mice (Figure S6), which have reduced amounts of functional innate immune cells, including Gr-1$^+$ cells,[39] and corroborated by subsequent *in vitro* assays demonstrating enhanced migration of human Tregs toward Gr-1$^+$ murine neutrophils and monocytes (Figure 6B). We further confirmed that human Tregs were responsive to murine CCL17/CCL22 (Figure 6A), and our results are in line with previous data.[65] Our finding that murine Gr-1$^+$ cell depletion resulted in delayed Treg detection at the human transplant (~3–4 weeks of delay; Figures 4 and 5) further suggested a major role of the CCR4-CCL17/CCL22 axis and warrants future investigations. Although use of human Tregs in mice could have been a limitation in understanding the migration of human Tregs, our results further demonstrated

that human Tregs are attracted *in vitro* and *in vivo* by murine Gr-1$^+$ cells. Notably, the work presented here in the context of transplantation suggests that, for Tregs that lack antigen specificity, the speed of migration to the transplant is controlled by the level of inflammation. In contrast, if Tregs present with donor-specificity, then their speed of migration is dictated by recognition of the donor antigen on endothelial cells, and they have been shown to migrate to the respective skin graft within a week, even in the absence of innate immune cells.[25,38] This is in agreement with the important role of antigen recognition on endothelial cells for recruitment of T cells into tissue that has been reported previously.[66] Recently, we extended this observation to CAR-expressing Tregs in the same humanized mouse model described in this study. Recognition of human leukocyte antigen (HLA)-A2 by A2-specific CAR Tregs favored migration of these cells to human skin.[38]

The results of our study demonstrate that NIS is a suitable radionuclide reporter gene for non-invasive long-term tracking of Tregs. The Treg therapy field is currently moving toward CAR Tregs, not least supported by several newly formed companies concerned with CAR development and optimization specifically for CAR Treg therapy. Importantly, like for anti-cancer CAR T cell therapy intended to treat tumors, there is also a requirement to monitor (CAR) Treg distribution and fate in therapy recipients. Here we provide a preclinical proof of principle to support translation of long-term tracking of (CAR) Tregs for future clinical application. Our methodology is well suited to support future CAR Treg tracking work because it relies on genetic engineering, which is a prerequisite for CAR Treg therapy, and therefore no major additional regulatory burden is added to enable imaging/monitoring. Further steps on the path to clinical translation include first convincing others that the added cost of including imaging in trials is low compared with the production cost and value of this cell therapy type and second combining CAR and the NIS reporter into the same final vector for human cell therapy. Besides aspects relating to clinical translation of Treg therapy, our work provides a strategy to implement quantitative 3D tomographic imaging into the workflows of preclinical Treg therapy development, and, not least, we contribute, with our findings regarding the role of innate immune cells, to the increasing understanding of human Treg migration to human skin grafts.

MATERIALS AND METHODS

Information regarding lentivirus production, flow cytometry marker analysis and fluorescence microscopy of cells, radiotracer uptake into cells, and determination of detection sensitivity of cells is detailed in the Supplemental Materials and Methods.

Ethics statement

The study involved human samples as well as animals. All procedures related to animal work were performed in accordance with all legal, ethical, and institutional requirements (PPL70/7302). Sample sizes of *in vivo* experiments were based on prior work from our laboratories[27,28,38] and kept to a minimum in line with law and ethical guidelines for animal research in the United Kingdom, as were the *in vivo* endpoints. Human skin and blood were obtained with ethics approval (institutional review board reference 09/H0707/86). Transplant-bearing animals were randomized before PBMC/Treg administration.

Reagents

Standard reagents were from Corning, Merck, Sarstedt, Thermo Fisher Scientific, TPP, or VWR. $[^{99m}Tc]TcO_4^-$ was generator-eluted as a sodium salt solution and used within 12 h of elution.

Animals

BRG mice (from A. Hayday, King's College London) were bred at licensed in-house animal facilities. NSG and C57BL/6 mice were purchased from Charles River Laboratories UK. All mice used were male and between 6 and 8 weeks old at the beginning of the experiment. Mice were maintained within the King's College London Biological Services Unit under specific pathogen-free conditions in a dedicated and licensed air-conditioned animal room (at 23°C ± 2°C and 40%–60% relative humidity) under light/dark cycles lasting 12 h every day. They were kept in individually ventilated standard plastic cages (IVCs; 501 cm^2 floor space, Tecniplast) including environmental enrichment and bedding material in the form of sterilized wood chips, paper strips, and one cardboard roll per cage. Maximum cage occupancy was five animals, and animals were moved to fresh cages with fresh environmental enrichment and bedding material twice per week. Sterilized tap water and food were available *ad libitum*; food was PicoLab Rodent Diet 20 (LabDiet) in the form of 2.5 × 1.6 × 1.0 cm oval pellets that were supplied at the top of the cages. For imaging, animals were anesthetized using isoflurane (1.5% [v/v] in pure O_2). After imaging, mice were left to recover from anesthesia (by withdrawal of anesthetic) in a pre-warmed chamber or sacrificed under anesthesia by cervical dislocation. No adverse events were associated with the procedures performed in this study, and animals put on weight in line with strain expectations (data from Charles River UK) throughout. Sentinel animals were kept on the same IVC racks as experimental animals and were confirmed to be healthy after completion of the studies.

Purification and culture of human PBMCs, Tregs, and Teffs

Cells were isolated from peripheral blood donated by anonymous healthy volunteers via the National Blood Service with informed consent according to our GMP protocols.[13–15] PBMCs were separated by Lymphoprep density gradient centrifugation (PAA Laboratories). CD4$^+$ T cells were enriched using RosetteSep (STEMCELL Technologies), followed by selection and separation of CD4$^+$CD25$^+$ Tregs and CD4$^+$CD25$^-$ Teffs using CD25 microbeads (Miltenyi Biotec). PBMCs were also depleted of CD25$^+$ cells by using CD25 microbeads (Miltenyi) and are referred to hereafter as CD25$^-$ PBMCs. Aliquots of CD25$^-$ PBMCs, Tregs, and Teffs were cryopreserved in 5% (v/v) human serum (BioSera) containing 10% (v/v) DMSO. Tregs were activated with anti-CD3/CD28 beads (1:1 bead:cell ratio, Miltenyi) and cultured in X-Vivo15 medium (Lonza) supplemented with 5% (v/v) human AB serum (BioSera; Treg medium) and 100 nM rapamycin (LC-Laboratories). 1,000 U/mL recombinant human IL-2

(Proleukin-Novartis) was added 72 h after cell isolation. After 10 days, Tregs were activated with anti-CD3/CD28 beads (1:1 bead:-cell ratio) for further expansion, which was repeated every 10 days for expansion and longevity. X-Vivo15 medium supplemented with human IL-2 and rapamycin (see above) was used to feed the cells every twodays. Tregs were cultured at 37°C in the presence of 5% (v/v) CO_2 in a humidified incubator.

Generation of NIS-GFP-expressing Tregs

Tregs (3 days after isolation) were transduced with lentiviral particles transferring NIS-GFP as described previously.[28,38] Briefly, tissue culture plates were coated with 50 μg/μL retronectin (TakaraBio) overnight at 4°C before concentrated lentivirus particles and Tregs were added. Treg medium contained anti-CD3/CD28 beads, IL-2, and rapamycin. After 72 h, virus-containing medium was washed off, and Tregs were maintained with fresh Treg medium every 48 h for seven days until fluorescence-activated cell sorting (FACS; BD FACSAria II, GFP channel). After sorting, NIS-GFP$^+$ Tregs were re-stimulated in Treg medium containing anti-CD3/CD28 beads, IL-2, and rapamycin.

Suppression assay

10^7 cells/mL HLA-A2-mismatched CD4$^+$CD25$^-$ Teffs were resuspended in staining solution containing 5 μM CellTrace Violet (CT-V) and incubated at 37°C for 15 min prior to co-culture. Tregs and labeled Teffs were co-cultured in X-Vivo medium at the indicated ratios in the presence of anti-CD3/CD28 beads. Teff proliferation was assessed after five days by quantifying cellular CT-V using flow cytometry. Results are shown as percent suppression (the inverse of percent Teff proliferation) relative to Teffs cultured alone.

Transwell migration assay

Female 8- to 10-week-old C57BL/6 mice received 1 mg/mL of zymosan intraperitoneally (i.p.) and were culled 4 and 18 h later to obtain neutrophils and monocytes from peritoneal cavities (via PBS washes), respectively. For monocytes, cells were left for 1 h at 37°C in RPMI medium without serum, allowing monocytes to adhere. Non-adherent cells were discarded. Cells were harvested, washed twice in cold PBS, counted, resuspended (RPMI medium containing 2% [v/v] fetal calf serum [FCS]), and 3×10^5 cells/700 μL were plated per 24 wells, forming the bottom wells of the migration assay. Some wells received admixtures of CCL17 and CCL22 (PeproTech, 1 ng/mL each).

Human Tregs were isolated/purified as described above. Prior to plating, some Tregs were treated for 1 h with 0.1 ng/mL PTX. Tregs were counted, resuspended at 3×10^5 Tregs/300μL in Treg medium and added to the top of the Transwell chambers (3-μm pore size). The Tregs that migrated to the bottom chamber within 2 h were counted while discriminating potentially present floating neutrophils or monocytes based on cell size.

Human skin xenograft transplant model

Human skin was obtained from routine abdominoplasty with informed consent and ethical approval (reference 06/Q0704/18). Donor HLA-A2$^+$ split-thickness skin grafts were transplanted onto 10- to 12-week-old recipient BRG or NSG mice as described previously.[7] Briefly, split-thickness (500–700 μm) skin explants were harvested with a dermatome (Zimmer), and ~1.5-cm^2 pieces were transplanted into graft beds on the indicated animals. Human skin grafts were fixed, epidermal side up, onto similarly sized defects on recipient backs with an absorbable tissue seal (Vet-Bond, Braun Medical). Grafts were covered with Fucidin microbicide (Leo Laboratories), secured with Tegaderm film dressing (3M) for seven days, and skin was allowed to engraft for 40 days in total before initiation of experiments.

5–6 weeks after skin transplantation, 5×10^6 PBMCs that were depleted of CD25$^+$ cells (see above) were then administered i.v. with or without 5×10^6 Tregs. No animals developed symptoms of xenogeneic GvHD, which was confirmed by maintenance of stable body weight. Skin grafts were subjected to visual and tactile inspection weekly for evidence of graft injury, and experiments ended 5–6 weeks after Teff transfer at the latest. As indicated in the experimental schemes, some BRG mice received 100 μg anti-mouse Gr-1 (RB6-8C5, Bio X Cell, 1 mg/mL in saline) i.p. every two days (Figure 3A).[38]

In vivo imaging and image analysis

Mice were anesthetized with 2% (v/v) isoflurane/O_2. SPECT/CT imaging was performed on animals that had received NIS-GFP$^+$ Tregs. 20 MBq [^{99m}Tc]TcO_4^- (in 100 μL sterile PBS) was administered i.v. into anaesthetized animals, and SPECT scans were acquired 40 min later (NanoScan SPECT/CT from Mediso equipped with high-resolution multi-pinhole collimators for mouse imaging; ^{99m}Tc SPECT resolution, ~0.7 mm; CT resolution, sub-100 μm isotropic voxel). CT (55 kVp tube voltage, 1,200 ms exposure time, 360 projections) was performed after tracer administration but before SPECT. Repeat imaging with the same radiotracer was not affected by tracer amounts from prior imaging sessions.[36]

SPECT/CT data were reconstructed using Tera-Tomo (Mediso) with corrections for attenuation, detector dead time, and radioisotope decay in place as needed. All images were analyzed using VivoQuant software (inviCRO), enabling delineation of regions of interest (ROIs) for quantification of activity. CT images were used to draw ROIs and provide the volumes required for standard uptake value calculations. The total activity in the whole animal (excluding the tail) at the time of tracer administration was defined as the injected dose (ID).

Ex vivo analyses of tissues

For analysis of radioactivity in harvested tissues, tissues were weighed, and radioactivity was quantified using a γ- counter (1282-Compugamma, LKB-Wallac) together with calibration standards. Data were expressed as %ID/g.

Skin grafts were harvested alongside normal mouse skin of similar size (from the lower back) for histology and frozen in Optimal Cutting Temperature (OCT) compound followed by tissue sectioning (8 μm). Thawed sections were fixed in 4% paraformaldehyde/PBS for 10 min, permeabilized (0.2% [v/v] Triton X-100/PBS), washed,

and blocked (20% [v/v] goat serum/PBS containing 0.1% fish skin gelatin and 0.25% [v/v] Tween 20) before being stained with the following primary antibodies (2 μg/mL) overnight at 4°C: anti-human CD3 (polyclonal rabbit, A0452, Dako), anti-FOXP3 (monoclonal rat, clone PCH101, eBioscience), anti-Gr-1 (monoclonal rat, RB6-8C5, eBioscience), and anti-GFP (monoclonal mouse, 3E6, Invitrogen). After washing, sections were stained with secondary antibodies: goat anti-mouse-AlexaFluor 555, anti-rabbit-AlexaFluor 568 (Invitrogen), and anti-rat-Cy5 (Jackson ImmunoResearch). Nuclei were stained with 4,6-diamidino-2-phenylindole (DAPI; 10 μg/mL PBS). Samples were mounted using fluorescence mounting medium (Dako) and imaged on a NikonA1 confocal microscope. Fiji/ImageJ v.1.5 software was used for analysis.

Statistical analysis

Statistical analysis was performed using Prism v.7 software (GraphPad); details are given in the text and figure legends. Generally, the significance level α was set to 0.05.

SUPPLEMENTAL INFORMATION

Supplemental Information can be found online at https://doi.org/10.1016/j.omtm.2020.12.003.

ACKNOWLEDGMENTS

We thank Drs. D. Boardman, J. Bordoloi, and E. Skourti for technical assistance and F. Laniyan for help with animal husbandry and logistics. This work was supported by the British Heart Foundation (RG/13/12/30395), the MRC Centre for Transplantation at King's College London (MR/J006742/1), Cancer Research UK (C48390/A21153), and the Wellcome/EPSRC Centre for Medical Engineering (WT203148/Z/16/Z). This research was funded/supported by the National Institute for Health Research (NIHR) Biomedical Research Centre based at Guy's and St Thomas' NHS Foundation Trust and King's College London and/or the NIHR Clinical Research Facility. The views expressed are those of the author(s) and not necessarily those of the NHS, the NIHR, or the Department of Health.

AUTHOR CONTRIBUTIONS

Conceptualization, F.M.M-B., G.L., G.O.F., L.A.S., and R.I.L.; Data Curation, J.J., S.N., G.O.F., and G.L.; Formal Analysis, A.V., J.J., Q.P., S.L.T., S.N., and Y.R.M.; Funding Acquisition, G.L. and G.O.F.; Investigation, A.V., C.S., J.J., L.A.S., Q.P., R.F.H., S.L.T., S.N., and Y.R.M.; Methodology, A.V., C.S., L.A.S., and S.N.; Project Administration, L.A.S., G.O.F., and G.L.; Resources, L.A.S., R.F.H., and Q.P.; Supervision, G.L., G.O.F., and L.A.S.; Validation, J.J., Q.P., S.L.T., and S.N.; Visualization, G.O.F. and J.J.; Writing – Original Draft, G.L. and G.O.F.; Writing – Review & Editing, all authors.

DECLARATION OF INTERESTS

G.L. is a co-founder of Quell Therapeutics. S.L.T. and Y.R.M. are employed by Quell Therapeutics (all data contributed by S.L.T. and Y.R.M. were collected before being employed by Quell Therapeutics).

REFERENCES

1. Katoh, H., Zheng, P., and Liu, Y. (2013). FOXP3: genetic and epigenetic implications for autoimmunity. J. Autoimmun. *41*, 72–78.
2. Miyara, M., Gorochov, G., Ehrenstein, M., Musset, L., Sakaguchi, S., and Amoura, Z. (2011). Human FoxP3+ regulatory T cells in systemic autoimmune diseases. Autoimmun. Rev. *10*, 744–755.
3. Siu, J.H.Y., Surendrakumar, V., Richards, J.A., and Pettigrew, G.J. (2018). T cell Allorecognition Pathways in Solid Organ Transplantation. Front. Immunol. *9*, 2548.
4. Krystufkova, E., Sekerkova, A., Striz, I., Brabcova, I., Girmanova, E., and Viklicky, O. (2012). Regulatory T cells in kidney transplant recipients: the effect of induction immunosuppression therapy. Nephrol. Dial. Transplant. *27*, 2576–2582.
5. Louis, S., Braudeau, C., Giral, M., Dupont, A., Moizant, F., Robillard, N., Moreau, A., Soulillou, J.P., and Brouard, S. (2006). Contrasting CD25hiCD4+T cells/FOXP3 patterns in chronic rejection and operational drug-free tolerance. Transplantation *81*, 398–407.
6. Krustrup, D., Madsen, C.B., Iversen, M., Engelholm, L., Ryder, L.P., and Andersen, C.B. (2013). The number of regulatory T cells in transbronchial lung allograft biopsies is related to FoxP3 mRNA levels in bronchoalveolar lavage fluid and to the degree of acute cellular rejection. Transpl. Immunol. *29*, 71–75.
7. Sagoo, P., Ali, N., Garg, G., Nestle, F.O., Lechler, R.I., and Lombardi, G. (2011). Human regulatory T cells with alloantigen specificity are more potent inhibitors of alloimmune skin graft damage than polyclonal regulatory T cells. Sci. Transl. Med. *3*, 83ra42.
8. Putnam, A.L., Safinia, N., Medvec, A., Laszkowska, M., Wray, M., Mintz, M.A., Trotta, E., Szot, G.L., Liu, W., Lares, A., et al. (2013). Clinical grade manufacturing of human alloantigen-reactive regulatory T cells for use in transplantation. Am. J. Transplant. *13*, 3010–3020.
9. Trzonkowski, P., Bieniaszewska, M., Juścińska, J., Dobyszuk, A., Krzystyniak, A., Marek, N., Myśliwska, J., and Hellmann, A. (2009). First-in-man clinical results of the treatment of patients with graft versus host disease with human ex vivo expanded CD4+CD25+CD127- T regulatory cells. Clin. Immunol. *133*, 22–26.
10. Brunstein, C.G., Miller, J.S., Cao, Q., McKenna, D.H., Hippen, K.L., Curtsinger, J., Defor, T., Levine, B.L., June, C.H., Rubinstein, P., et al. (2011). Infusion of ex vivo expanded T regulatory cells in adults transplanted with umbilical cord blood: safety profile and detection kinetics. Blood *117*, 1061–1070.
11. Marek-Trzonkowska, N., Mysliwiec, M., Dobyszuk, A., Grabowska, M., Techmanska, I., Juscinska, J., Wujtewicz, M.A., Witkowski, P., Mlynarski, W., Balcerska, A., et al. (2012). Administration of CD4+CD25highCD127- regulatory T cells preserves β-cell function in type 1 diabetes in children. Diabetes Care *35*, 1817–1820.
12. Bluestone, J.A., Buckner, J.H., Fitch, M., Gitelman, S.E., Gupta, S., Hellerstein, M.K., Herold, K.C., Lares, A., Lee, M.R., Li, K., et al. (2015). Type 1 diabetes immunotherapy using polyclonal regulatory T cells. Sci. Transl. Med. *7*, 315ra189.
13. Safinia, N., Vaikunthanathan, T., Fraser, H., Thirkell, S., Lowe, K., Blackmore, L., Whitehouse, G., Martinez-Llordella, M., Jassem, W., Sanchez-Fueyo, A., et al. (2016). Successful expansion of functional and stable regulatory T cells for immunotherapy in liver transplantation. Oncotarget *7*, 7563–7577.
14. Afzali, B., Edozie, F.C., Fazekasova, H., Scottà, C., Mitchell, P.J., Canavan, J.B., Kordasti, S.Y., Chana, P.S., Ellis, R., Lord, G.M., et al. (2013). Comparison of regulatory T cells in hemodialysis patients and healthy controls: implications for cell therapy in transplantation. Clin. J. Am. Soc. Nephrol. *8*, 1396–1405.
15. Sawitzki, B., Harden, P.N., Reinke, P., Moreau, A., Hutchinson, J.A., Game, D.S., Tang, Q., Guinan, E.C., Battaglia, M., Burlingham, W.J., et al. (2020). Regulatory cell therapy in kidney transplantation (The ONE Study): a harmonised design and analysis of seven non-randomised, single-arm, phase 1/2A trials. Lancet *395*, 1627–1639.
16. Ashmore-Harris, C., Iafrate, M., Saleem, A., and Fruhwirth, G.O. (2020). Non-invasive Reporter Gene Imaging of Cell Therapies, including T Cells and Stem Cells. Mol. Ther. *28*, 1392–1416.
17. Sharif-Paghaleh, E., Leech, J., Sunassee, K., Ali, N., Sagoo, P., Lechler, R.I., Smyth, L.A., Lombardi, G., and Mullen, G.E. (2014). Monitoring the efficacy of dendritic cell vaccination by early detection of (99m) Tc-HMPAO-labelled CD4(+) T cells. Eur. J. Immunol. *44*, 2188–2191.

18. Roca, M., de Vries, E.F., Jamar, F., Israel, O., and Signore, A.; Inflammation/Infection Taskgroup of the European Association of Nuclear Medicine (2010). Guidelines for the labelling of leucocytes with (111)In-oxine. Eur. J. Nucl. Med. Mol. Imaging *37*, 835–841.
19. Weist, M.R., Starr, R., Aguilar, B., Chea, J., Miles, J., Poku, E., Gerdts, E., Yang, X., Priceman, S., Forman, S., et al. (2018). Positron emission tomography of adoptively transferred chimeric antigen receptor T cells with Zirconium-89 oxine. J. Nucl. Med. *59*, 1531–1537.
20. Man, F., Lim, L., Volpe, A., Gabizon, A., Shmeeda, H., Draper, B., Parente-Pereira, A.C., Maher, J., Blower, P.J., Fruhwirth, G.O., et al. (2019). In Vivo PET Tracking of (89)Zr-Labeled Vgamma9Vdelta2 T Cells to Mouse Xenograft Breast Tumors Activated with Liposomal Alendronate. Mol. Ther *27*, 219–229.
21. Sato, N., Wu, H., Asiedu, K.O., Szajek, L.P., Griffiths, G.L., and Choyke, P.L. (2015). (89)Zr-Oxine Complex PET Cell Imaging in Monitoring Cell-based Therapies. Radiology *275*, 490–500.
22. Iafrate, M., and Fruhwirth, G.O. (2020). How Non-invasive *in vivo* Cell Tracking Supports the Development and Translation of Cancer Immunotherapies. Front. Physiol. *11*, 154.
23. Suffner, J., Hochweller, K., Kühnle, M.C., Li, X., Kroczek, R.A., Garbi, N., and Hämmerling, G.J. (2010). Dendritic cells support homeostatic expansion of Foxp3+ regulatory T cells in Foxp3.LuciDTR mice. J. Immunol. *184*, 1810–1820.
24. Nguyen, V.H., Zeiser, R., Dasilva, D.L., Chang, D.S., Beilhack, A., Contag, C.H., and Negrin, R.S. (2007). In vivo dynamics of regulatory T-cell trafficking and survival predict effective strategies to control graft-versus-host disease following allogeneic transplantation. Blood *109*, 2649–2656.
25. Dawson, N.A., Lamarche, C., Hoeppli, R.E., Bergqvist, P., Fung, V.C., McIver, E., Huang, Q., Gillies, J., Speck, M., Orban, P.C., et al. (2019). Systematic testing and specificity mapping of alloantigen-specific chimeric antigen receptors in regulatory T cells. JCI Insight *4*, 4.
26. Keu, K.V., Witney, T.H., Yaghoubi, S., Rosenberg, J., Kurien, A., Magnusson, R., Williams, J., Habte, F., Wagner, J.R., Forman, S., et al. (2017). Reporter gene imaging of targeted T cell immunotherapy in recurrent glioma. Sci. Transl. Med. *9*, eaag2196.
27. Fruhwirth, G.O., Diocou, S., Blower, P.J., Ng, T., and Mullen, G.E. (2014). A whole-body dual-modality radionuclide optical strategy for preclinical imaging of metastasis and heterogeneous treatment response in different microenvironments. J. Nucl. Med. *55*, 686–694.
28. Volpe, A., Man, F., Lim, L., Khoshnevisan, A., Blower, J., Blower, P.J., and Fruhwirth, G.O. (2018). Radionuclide-fluorescence Reporter Gene Imaging to Track Tumor Progression in Rodent Tumor Models. J. Vis. Exp. *133*, e57088.
29. Ashmore-Harris, C., Blackford, S.J., Grimsdell, B., Kurtys, E., Glatz, M.C., Rashid, T.S., and Fruhwirth, G.O. (2019). Reporter gene-engineering of human induced pluripotent stem cells during differentiation renders in vivo traceable hepatocyte-like cells accessible. Stem Cell Res. (Amst.) *41*, 101599.
30. Kang, J.H., Lee, D.S., Paeng, J.C., Lee, J.S., Kim, Y.H., Lee, Y.J., Hwang, D.W., Jeong, J.M., Lim, S.M., Chung, J.K., and Lee, M.C. (2005). Development of a sodium/iodide symporter (NIS)-transgenic mouse for imaging of cardiomyocyte-specific reporter gene expression. J. Nucl. Med. *46*, 479–483.
31. Sharif-Paghaleh, E., Sunassee, K., Tavaré, R., Ratnasothy, K., Koers, A., Ali, N., Alhabbab, R., Blower, P.J., Lechler, R.I., Smyth, L.A., et al. (2011). In vivo SPECT reporter gene imaging of regulatory T cells. PLoS ONE *6*, e25857.
32. Volpe, A., Lang, C., Lim, L., Man, F., Kurtys, E., Ashmore-Harris, C., Johnson, P., Skourti, E., de Rosales, R.T.M., and Fruhwirth, G.O. (2020). Spatiotemporal PET imaging reveals differences in CAR-T tumour retention in triple-negative breast cancer models. Mol. Ther. *28*, 2271–2285.
33. O'Doherty, J., Jauregui-Osoro, M., Brothwood, T., Szyszko, T., Marsden, P.K., O'Doherty, M.J., Cook, G.J.R., Blower, P.J., and Lewington, V. (2017). ^{18}F-Tetrafluoroborate, a PET Probe for Imaging Sodium/Iodide Symporter Expression: Whole-Body Biodistribution, Safety, and Radiation Dosimetry in Thyroid Cancer Patients. J. Nucl. Med. *58*, 1666–1671.
34. Meller, J., and Becker, W. (2002). The continuing importance of thyroid scintigraphy in the era of high-resolution ultrasound. Eur. J. Nucl. Med. Mol. Imaging *29* (*Suppl 2*), S425–S438.
35. Moser, B., Loetscher, M., Piali, L., and Loetscher, P. (1998). Lymphocyte responses to chemokines. Int. Rev. Immunol. *16*, 323–344.
36. Diocou, S., Volpe, A., Jauregui-Osoro, M., Boudjemeline, M., Chuamsaamarkkee, K., Man, F., Blower, P.J., Ng, T., Mullen, G.E.D., and Fruhwirth, G.O. (2017). [^{18}F]tetrafluoroborate-PET/CT enables sensitive tumor and metastasis in vivo imaging in a sodium iodide symporter-expressing tumor model. Sci. Rep. *7*, 946.
37. Portulano, C., Paroder-Belenitsky, M., and Carrasco, N. (2014). The Na+/I- symporter (NIS): mechanism and medical impact. Endocr. Rev. *35*, 106–149.
38. Boardman, D.A., Philippeos, C., Fruhwirth, G.O., Ibrahim, M.A., Hannen, R.F., Cooper, D., Marelli-Berg, F.M., Watt, F.M., Lechler, R.I., Maher, J., et al. (2017). Expression of a Chimeric Antigen Receptor Specific for Donor HLA Class I Enhances the Potency of Human Regulatory T Cells in Preventing Human Skin Transplant Rejection. Am. J. Transplant. *17*, 931–943.
39. Kenney, L.L., Shultz, L.D., Greiner, D.L., and Brehm, M.A. (2016). Humanized Mouse Models for Transplant Immunology. Am. J. Transplant. *16*, 389–397.
40. Iellem, A., Mariani, M., Lang, R., Recalde, H., Panina-Bordignon, P., Sinigaglia, F., and D'Ambrosio, D. (2001). Unique chemotactic response profile and specific expression of chemokine receptors CCR4 and CCR8 by CD4(+)CD25(+) regulatory T cells. J. Exp. Med. *194*, 847–853.
41. Ishida, T., and Ueda, R. (2006). CCR4 as a novel molecular target for immunotherapy of cancer. Cancer Sci. *97*, 1139–1146.
42. Blat, D., Zigmond, E., Alteber, Z., Waks, T., and Eshhar, Z. (2014). Suppression of murine colitis and its associated cancer by carcinoembryonic antigen-specific regulatory T cells. Mol. Ther. *22*, 1018–1028.
43. Fransson, M., Piras, E., Burman, J., Nilsson, B., Essand, M., Lu, B., Harris, R.A., Magnusson, P.U., Brittebo, E., and Loskog, A.S. (2012). CAR/FoxP3-engineered T regulatory cells target the CNS and suppress EAE upon intranasal delivery. J. Neuroinflammation *9*, 112.
44. MacDonald, K.G., Hoeppli, R.E., Huang, Q., Gillies, J., Luciani, D.S., Orban, P.C., Broady, R., and Levings, M.K. (2016). Alloantigen-specific regulatory T cells generated with a chimeric antigen receptor. J. Clin. Invest. *126*, 1413–1424.
45. Maude, S.L., Laetsch, T.W., Buechner, J., Rives, S., Boyer, M., Bittencourt, H., Bader, P., Verneris, M.R., Stefanski, H.E., Myers, G.D., et al. (2018). Tisagenlecleucel in Children and Young Adults with B-Cell Lymphoblastic Leukemia. N. Engl. J. Med. *378*, 439–448.
46. Neelapu, S.S., Locke, F.L., Bartlett, N.L., Lekakis, L.J., Miklos, D.B., Jacobson, C.A., Braunschweig, I., Oluwole, O.O., Siddiqi, T., Lin, Y., et al. (2017). Axicabtagene Ciloleucel CAR T-Cell Therapy in Refractory Large B-Cell Lymphoma. N. Engl. J. Med. *377*, 2531–2544.
47. Minn, I., Huss, D.J., Ahn, H.H., Chinn, T.M., Park, A., Jones, J., Brummet, M., Rowe, S.P., Sysa-Shah, P., Du, Y., et al. (2019). Imaging CAR T cell therapy with PSMA-targeted positron emission tomography. Sci. Adv. *5*, eaaw5096.
48. Cherry, S.R., Jones, T., Karp, J.S., Qi, J., Moses, W.W., and Badawi, R.D. (2018). Total-Body PET: Maximizing Sensitivity to Create New Opportunities for Clinical Research and Patient Care. J. Nucl. Med. *59*, 3–12.
49. Volpe, A., Lang, C., Lim, L., Man, F., Kurtys, E., Ashmore-Harris, C., Johnson, P., Skourti, E., de Rosales, R.T.M., and Fruhwirth, G.O. (2020). Spatiotemporal PET Imaging Reveals Differences in CAR-T Tumor Retention in Triple-Negative Breast Cancer Models. Mol. Ther. *28*, 2271–2285.
50. Khoshnevisan, A., Chuamsaamarkkee, K., Boudjemeline, M., Jackson, A., Smith, G.E., Gee, A.D., Fruhwirth, G.O., and Blower, P.J. (2017). 18F-Fluorosulfate for PET Imaging of the Sodium-Iodide Symporter: Synthesis and Biologic Evaluation In Vitro and In Vivo. J. Nucl. Med. *58*, 156–161.
51. Dohán, O., Portulano, C., Basquin, C., Reyna-Neyra, A., Amzel, L.M., and Carrasco, N. (2007). The Na+/I symporter (NIS) mediates electroneutral active transport of the environmental pollutant perchlorate. Proc. Natl. Acad. Sci. USA *104*, 20250–20255.
52. Wilkie, S., Burbridge, S.E., Chiapero-Stanke, L., Pereira, A.C., Cleary, S., van der Stegen, S.J., Spicer, J.F., Davies, D.M., and Maher, J. (2010). Selective expansion of chimeric antigen receptor-targeted T-cells with potent effector function using interleukin-4. J. Biol. Chem. *285*, 25538–25544.

53. Volpe, A., Kurtys, E., and Fruhwirth, G.O. (2018). Cousins at work: How combining medical with optical imaging enhances in vivo cell tracking. Int. J. Biochem. Cell Biol. *102*, 40–50.

54. Zhang, N., Schröppel, B., Lal, G., Jakubzick, C., Mao, X., Chen, D., Yin, N., Jessberger, R., Ochando, J.C., Ding, Y., and Bromberg, J.S. (2009). Regulatory T cells sequentially migrate from inflamed tissues to draining lymph nodes to suppress the alloimmune response. Immunity *30*, 458–469.

55. Chauhan, S.K., Saban, D.R., Dohlman, T.H., and Dana, R. (2014). CCL-21 conditioned regulatory T cells induce allotolerance through enhanced homing to lymphoid tissue. J. Immunol. *192*, 817–823.

56. Kumar, V., Patel, S., Tcyganov, E., and Gabrilovich, D.I. (2016). The Nature of Myeloid-Derived Suppressor Cells in the Tumor Microenvironment. Trends Immunol. *37*, 208–220.

57. Nadkarni, S., Smith, J., Sferruzzi-Perri, A.N., Ledwozyw, A., Kishore, M., Haas, R., Mauro, C., Williams, D.J., Farsky, S.H., Marelli-Berg, F.M., and Perretti, M. (2016). Neutrophils induce proangiogenic T cells with a regulatory phenotype in pregnancy. Proc. Natl. Acad. Sci. USA *113*, E8415–E8424.

58. Mishalian, I., Bayuh, R., Eruslanov, E., Michaeli, J., Levy, L., Zolotarov, L., Singhal, S., Albelda, S.M., Granot, Z., and Fridlender, Z.G. (2014). Neutrophils recruit regulatory T-cells into tumors via secretion of CCL17–a new mechanism of impaired antitumor immunity. Int. J. Cancer *135*, 1178–1186.

59. Dugast, A.S., Haudebourg, T., Coulon, F., Heslan, M., Haspot, F., Poirier, N., Vuillefroy de Silly, R., Usal, C., Smit, H., Martinet, B., et al. (2008). Myeloid-derived suppressor cells accumulate in kidney allograft tolerance and specifically suppress effector T cell expansion. J. Immunol. *180*, 7898–7906.

60. Nakamura, T., Nakao, T., Ashihara, E., and Yoshimura, N. (2016). Myeloid-derived Suppressor Cells Recruit CD4(+)/Foxp3(+) Regulatory T Cells in a Murine Cardiac Allograft. Transplant. Proc. *48*, 1275–1278.

61. Luan, Y., Mosheir, E., Menon, M.C., Wilson, D., Woytovich, C., Ochando, J., and Murphy, B. (2013). Monocytic myeloid-derived suppressor cells accumulate in renal transplant patients and mediate CD4(+) Foxp3(+) Treg expansion. Am. J. Transplant. *13*, 3123–3131.

62. Okano, S., Abu-Elmagd, K., Kish, D.D., Keslar, K., Baldwin, W.M., 3rd, Fairchild, R.L., Fujiki, M., Khanna, A., Osman, M., Costa, G., et al. (2018). Myeloid-derived suppressor cells increase and inhibit donor-reactive T cell responses to graft intestinal epithelium in intestinal transplant patients. Am. J. Transplant. *18*, 2544–2558.

63. Garcia, M.R., Ledgerwood, L., Yang, Y., Xu, J., Lal, G., Burrell, B., Ma, G., Hashimoto, D., Li, Y., Boros, P., et al. (2010). Monocytic suppressive cells mediate cardiovascular transplantation tolerance in mice. J. Clin. Invest. *120*, 2486–2496.

64. Augier, S., Ciucci, T., Luci, C., Carle, G.F., Blin-Wakkach, C., and Wakkach, A. (2010). Inflammatory blood monocytes contribute to tumor development and represent a privileged target to improve host immunosurveillance. J. Immunol. *185*, 7165–7173.

65. Santulli-Marotto, S., Boakye, K., Lacy, E., Wu, S.-J., Luongo, J., Kavalkovich, K., Coelho, A., Hogaboam, C.M., and Ryan, M. (2013). Engagement of two distinct binding domains on CCL17 is required for signaling through CCR4 and establishment of localized inflammatory conditions in the lung. PLoS ONE *8*, e81465.

66. Marelli-Berg, F.M., Frasca, L., Weng, L., Lombardi, G., and Lechler, R.I. (1999). Antigen recognition influences transendothelial migration of CD4+ T cells. J. Immunol. *162*, 696–703.

OMTM, Volume *20*

Supplemental Information

Spatiotemporal *in vivo* tracking of polyclonal human regulatory T cells (Tregs) reveals a role for innate immune cells in Treg transplant recruitment

Jacinta Jacob, Suchita Nadkarni, Alessia Volpe, Qi Peng, Sim L. Tung, Rosalind F. Hannen, Yasmin R. Mohseni, Cristiano Scotta, Federica M. Marelli-Berg, Robert I. Lechler, Lesley A. Smyth, Gilbert O. Fruhwirth, and Giovanna Lombardi

SUPPLEMENTARY INFORMATION

This supplement contains Supplementary Methods detailing lentivirus production, radiotracer uptake assays, flow cytometry and microscopy of cells, and the determination of detection limits of traceable Tregs. Moreover, it contains several Supplementary Figures, which are referred to in the main text.

Supplementary Methods

Lentivirus production. For lentivirus production we used HEK293T cells (ATCC, USA), which were cultured in DMEM supplemented with 10% (v/v) heat-inactivated fetal calf serum (FCS), 100 units/mL penicillin and streptomycin, and 2 mM *L*-glutamine. HEK293T cells were transfected with the target plasmid pLNT/SFFV NIS-GFP and the corresponding packaging plasmids pΔ8.91 and pVSV-G as previously described.[1] Virus-containing culture supernatant was harvested after 48h, concentrated four-fold using PEG-IT (System Biosciences) and stored at -80C until use.

Flow cytometric marker analysis. Cells were stained in phosphate-buffered saline (PBS) supplemented with 1%(v/v) FCS/5mM EDTA using fluorescently conjugated primary antibodies at concentrations ranging 2-5 μg/mL. Antibodies used were specific for CD4 (OKT4), FOXP3 (PCH101), HLA-A2 (BB7.2) (all from eBioscience); CD25 (clone 4E3 for assessment of Treg purity; clone 2A3 for all other experiments), CTLA-4 (BNI3) (all from BD Bioscience); CD127 (A019D5), CD62L (DREG-56), CLA (HECA-452) (all from BioLegend), and CCR4 (205410l; R&D Systems). Only live cells were analyzed with dead cells being excluded based on counterstaining with a near-infrared dye (LIVE/DEAD Fixable Near-IR Dead Cell Stain kit; Thermo Fisher). Intracellular staining was performed using the Fix/Perm kit (eBioscience). Data were acquired using a LSRFortessa II (BDBioscience) and analyzed using FlowJo v.7 (Tree Star).

Fluorescence microscopy of NIS-GFP$^+$ Tregs. NIS-GFP$^+$ Tregs were plated onto retronectin-coated translucent sterile 24-well dishes and subjected to life cell microscopy using a wide-field fluorescence microscope equipped with a filter cube suitable for GFP imaging (EVOS FL Cell Imaging System; Thermo Fisher, UK).

***In vitro* radio tracer uptake in NIS-GFP$^+$ Tregs.** To determine reporter gene function 10^6 cells (Tregs or NIS-GFP$^+$ Tregs) were transferred into Eppendorf tubes, washed with ice-cold PBS, and resuspended in 1 mL HBSS. 50kBq [^{99m}Tc]TcO_4^- was added and cells were incubated at 37°C for 30 min. To block NIS function, 10 µL of a 500 µM $NaClO_4$ solution was added 20 min prior to the assay. Cells were pelleted, supernatant was collected, and cells were washed three times with 1 mL PBS before being resuspended in growth medium for γ-counting and further culture. Cellular radiotracer uptake was determined by quantifying radioactivity in cells and pooled supernatant/wash solutions using a γ-counter (1282-Compugamma, LKB-Wallac) and calculated using Equ.S1 (wherein Cpm represents decay-corrected radioactivity counts per minute).

$$\%Radioactivity\ uptake = \frac{Cpm\ [Cells]}{Cpm\ [Cells] + Cpm\ [Supernatant\ and\ Washes]} \cdot 100 \qquad \text{(Equ.S1)}$$

For assays involving pre-labelling to subsequently determine radiolabeling effects *e.g.* phenotype, suppression etc., $10x10^6$ NIS-GFP$^+$ Tregs were incubated as above with 3MBq [^{99m}Tc]TcO_4^- resulting in typical cellular radioactivity levels of 78.4 ± 11.5 mBq/cell.

Determination of the minimum number of NIS-positive cells detectable by nanoSPECT/CT. To determine the detection sensitivity of NIS-GFP$^+$ Tregs in the nanoSPECT/CT scanner (Bioscan/Mediso, Silver Upgrade), we prepared cell pellets consisting of pre-labeled NIS-positive (86 ± 5.7 mBq [^{99m}Tc]TcO_4^-/cell) and NIS-negative untransduced Tregs. The total cell number per pellet was kept constant at 10^6 cells. This procedure has previously been used to determine the detection limit of reporter gene expressing cells in preclinical SPECT and PET instruments.[2, 3] Briefly, cell mixtures were prepared in 50 µL PBS$^{+/+}$ in Eppendorf Tubes, pelleted and immediately scanned in the nanoSPECT/CT equipment for 45 min. Reconstructed images were analyzed using Vivoquant whereby radioactivity was measured in volumes of interest drawn with the help of CT information (tube walls as boundaries). Measurements were performed in triplicate. In line with standard analytical procedures, we defined the limit of detection (LOD) to be 3-times the standard deviation above background signals (Fig.S4).

Supplementary References

1. Volpe, A, Man, F, Lim, L, Khoshnevisan, A, Blower, J, Blower, PJ, and Fruhwirth, GO. **Radionuclide-fluorescence Reporter Gene Imaging to Track Tumor Progression in Rodent Tumor Models**. *J Vis Exp*. 2018; **133**: e57088.
2. Fruhwirth, GO, Diocou, S, Blower, PJ, Ng, T, and Mullen, GE. **A whole-body dual-modality radionuclide optical strategy for preclinical imaging of metastasis and heterogeneous treatment response in different microenvironments**. *J Nucl Med*. 2014; **55**: 686-694.
3. Diocou, S, Volpe, A, Jauregui-Osoro, M, Boudjemeline, M, Chuamsaamarkkee, K, Man, F, Blower, PJ, Ng, T, Mullen, GED, and Fruhwirth, GO. **[(18)F]tetrafluoroborate-PET/CT enables sensitive tumor and metastasis in vivo imaging in a sodium iodide symporter-expressing tumor model**. *Sci Rep*. 2017; **7**: 946.
4. Dohan, O, Portulano, C, Basquin, C, Reyna-Neyra, A, Amzel, LM, and Carrasco, N. **The Na+/I symporter (NIS) mediates electroneutral active transport of the environmental pollutant perchlorate**. *Proc Natl Acad Sci U S A*. 2007; **104**: 20250-20255.
5. Khoshnevisan, A, Chuamsaamarkkee, K, Boudjemeline, M, Jackson, A, Smith, GE, Gee, AD, Fruhwirth, GO, and Blower, PJ. **18F-Fluorosulfate for PET Imaging of the Sodium-Iodide Symporter: Synthesis and Biologic Evaluation In Vitro and In Vivo**. *J Nucl Med*. 2017; **58**: 156-161.
6. Portulano, C, Paroder-Belenitsky, M, and Carrasco, N. **The Na+/I- symporter (NIS): mechanism and medical impact**. *Endocr Rev*. 2014; **35**: 106-149.
7. Paroder-Belenitsky, M, Maestas, MJ, Dohan, O, Nicola, JP, Reyna-Neyra, A, Follenzi, A, Dadachova, E, Eskandari, S, Amzel, LM, and Carrasco, N. **Mechanism of anion selectivity and stoichiometry of the Na+/I- symporter (NIS)**. *Proc Natl Acad Sci U S A*. 2011; **108**: 17933-17938.
8. Ashmore-Harris, C, Blackford, SJ, Grimsdell, B, Kurtys, E, Glatz, MC, Rashid, TS, and Fruhwirth, GO. **Reporter gene-engineering of human induced pluripotent stem cells during differentiation renders in vivo traceable hepatocyte-like cells accessible**. *Stem Cell Res*. 2019; **41**: 101599.
9. Man, F, Lim, L, Volpe, A, Gabizon, A, Shmeeda, H, Draper, B, Parente-Pereira, AC, Maher, J, Blower, PJ, Fruhwirth, GO*, et al.* **In Vivo PET Tracking of (89)Zr-Labeled Vgamma9Vdelta2 T Cells to Mouse Xenograft Breast Tumors Activated with Liposomal Alendronate**. *Molecular therapy : the journal of the American Society of Gene Therapy*. 2019; **27**: 219-229.
10. Volpe, A, Lang, C, Lim, L, Man, F, Kurtys, E, Ashmore-Harris, C, Johnson, P, Skourti, E, de Rosales, RTM, and Fruhwirth, GO. **Spatiotemporal PET Imaging Reveals Differences in CAR-T Tumor Retention in Triple-Negative Breast Cancer Models**. *Molecular therapy : the journal of the American Society of Gene Therapy*. 2020.

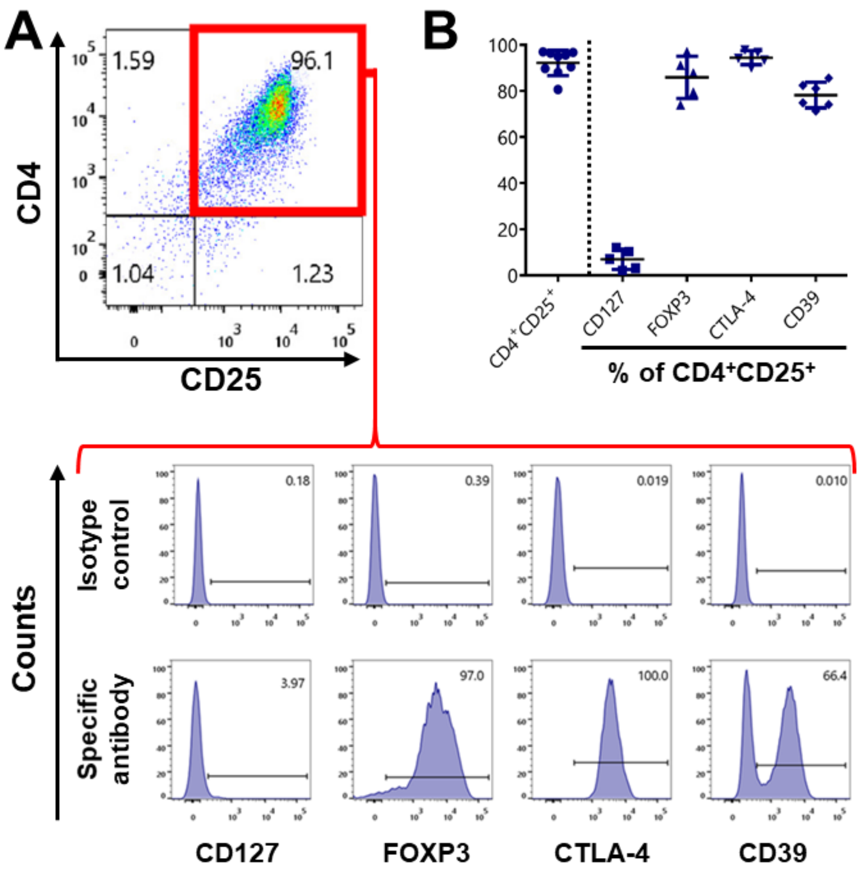

Figure S1. Quality of *in vitro* expanded human Tregs.| (A) Tregs were isolated from different human donors and expanded *in vitro* for 20 days. $CD4^{+}CD25^{+}$ population was analyzed for expression of CD127, FOXP3, CTLA-4 and CD39. **(B)** Cumulative data ($n \geq 5$) from individual donors indicating agreement with the expected phenotype for human Tregs; error bars represent SD.

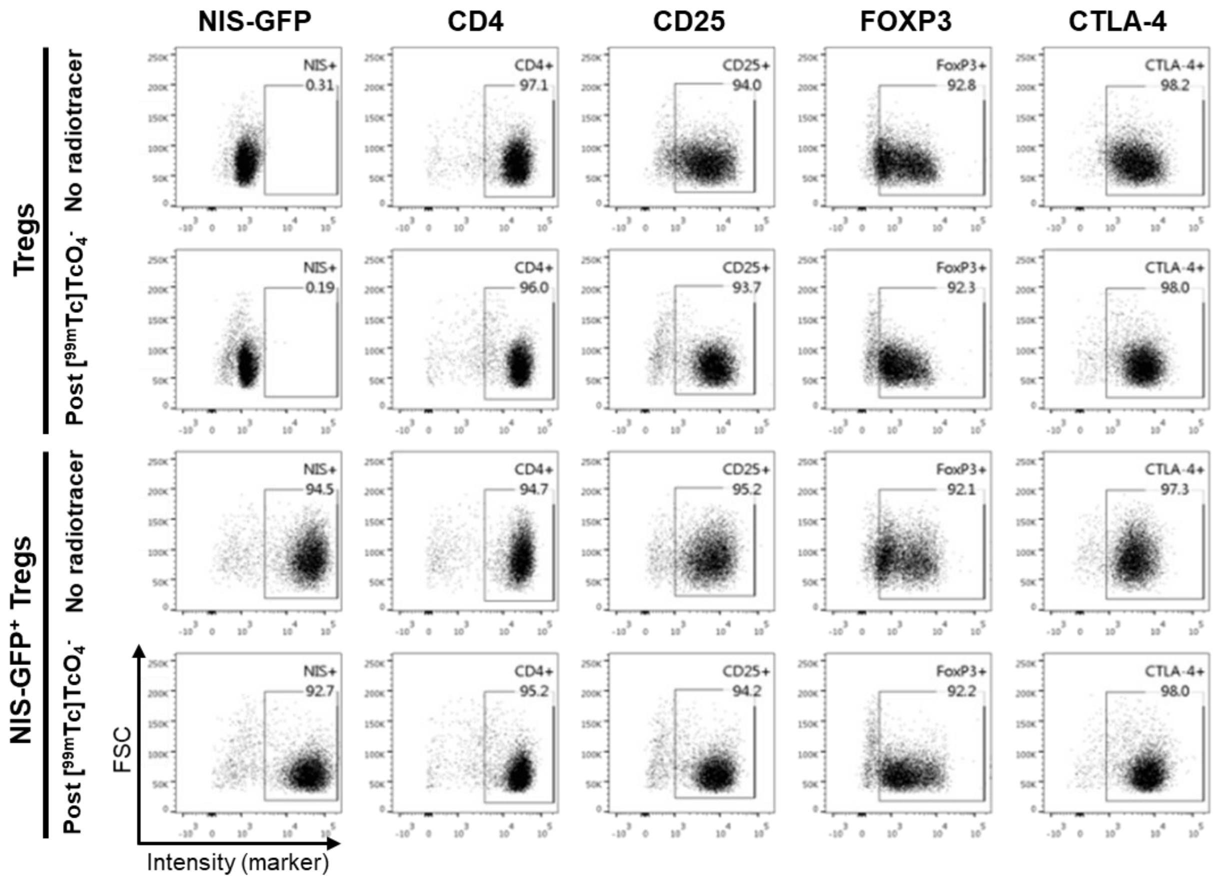

Figure S2. Representative individual data supplementing the marker panel analyses of Fig.2A-B.| Established Treg markers CD25, FOXP3 and CTLA-4 were quantified alongside CD4 (all *via* antibody staining) and NIS-GFP (*via* intrinsic GFP fluorescence). Tregs were analyzed 6 h (one half-life) after exposure to the NIS radiotracer [^{99m}Tc]TcO_4^-. Cumulative data describing the percentage and the mean fluorescence intensities of positive cells are shown in Fig.2A-B. Corresponding representative individual data are shown here.

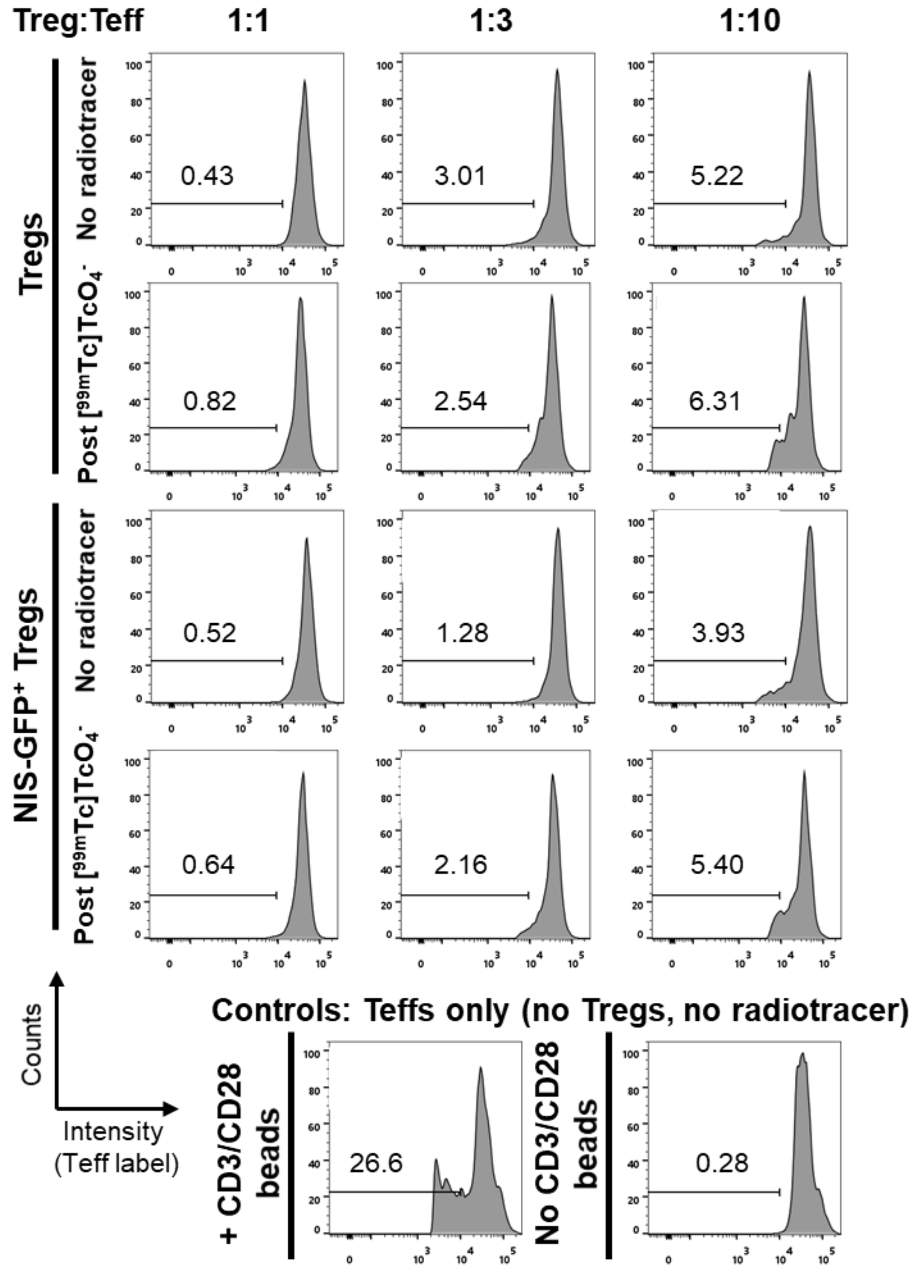

Figure S3. Representative individual data supplementing the Treg suppression analyses of Fig.2C.| Comparison of suppressive capacity of NIS-GFP^{+} Tregs and untransduced Tregs in the presence of absence of radioactivity as indicated. Inset numbers are absolute %Teffs that proliferated in the presence of CD3/CD28 beads with headers indicating the relative Treg:Teff ratios. Teffs cultured in the absence of Tregs proliferated readily (left bottom) and served for data normalization; unstimulated Teffs did not proliferate (right bottom). Representative data of *n*=3 experiments (*n* different donors). Cumulative data are shown in Fig.2C.

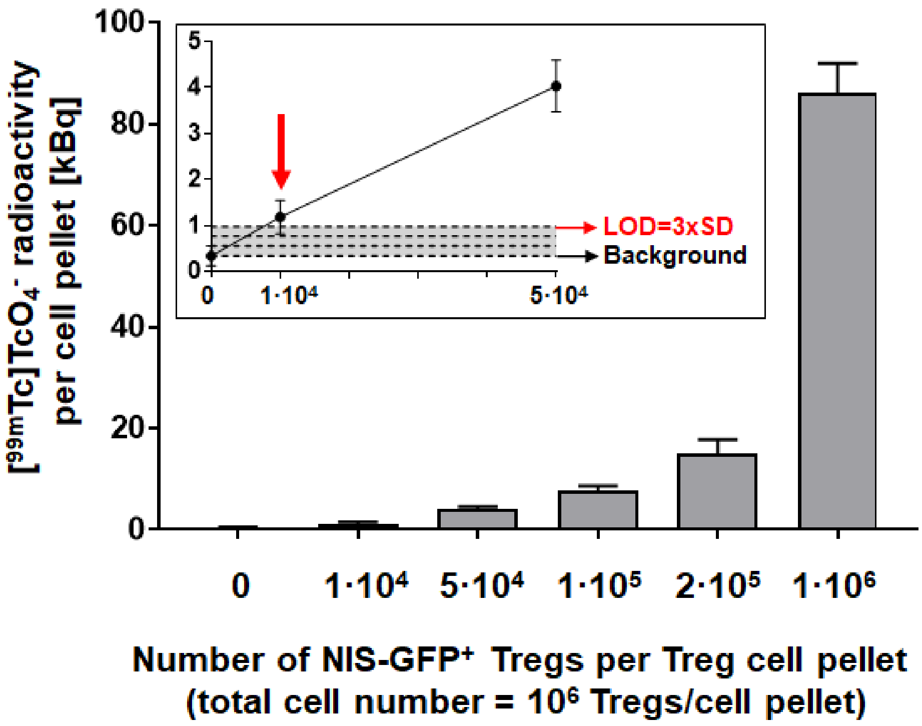

Figure S4. Determination of the detection sensitivity of NIS-GFP$^+$ Tregs.| NIS-GFP$^+$ Tregs were labeled with the radiotracer $^{99m}TcO_4^-$ and serially diluted into untransduced and unlabeled Tregs. Cell pellets in Eppendorf tubes were imaged and quantified using nanoSPECT (see Supplementary Methods). From triplicate measurements (error bars are SD), we determined the detection limit to be ~10,000 NIS-positive Tregs within a cell pellet of 10^6 untransduced Tregs.

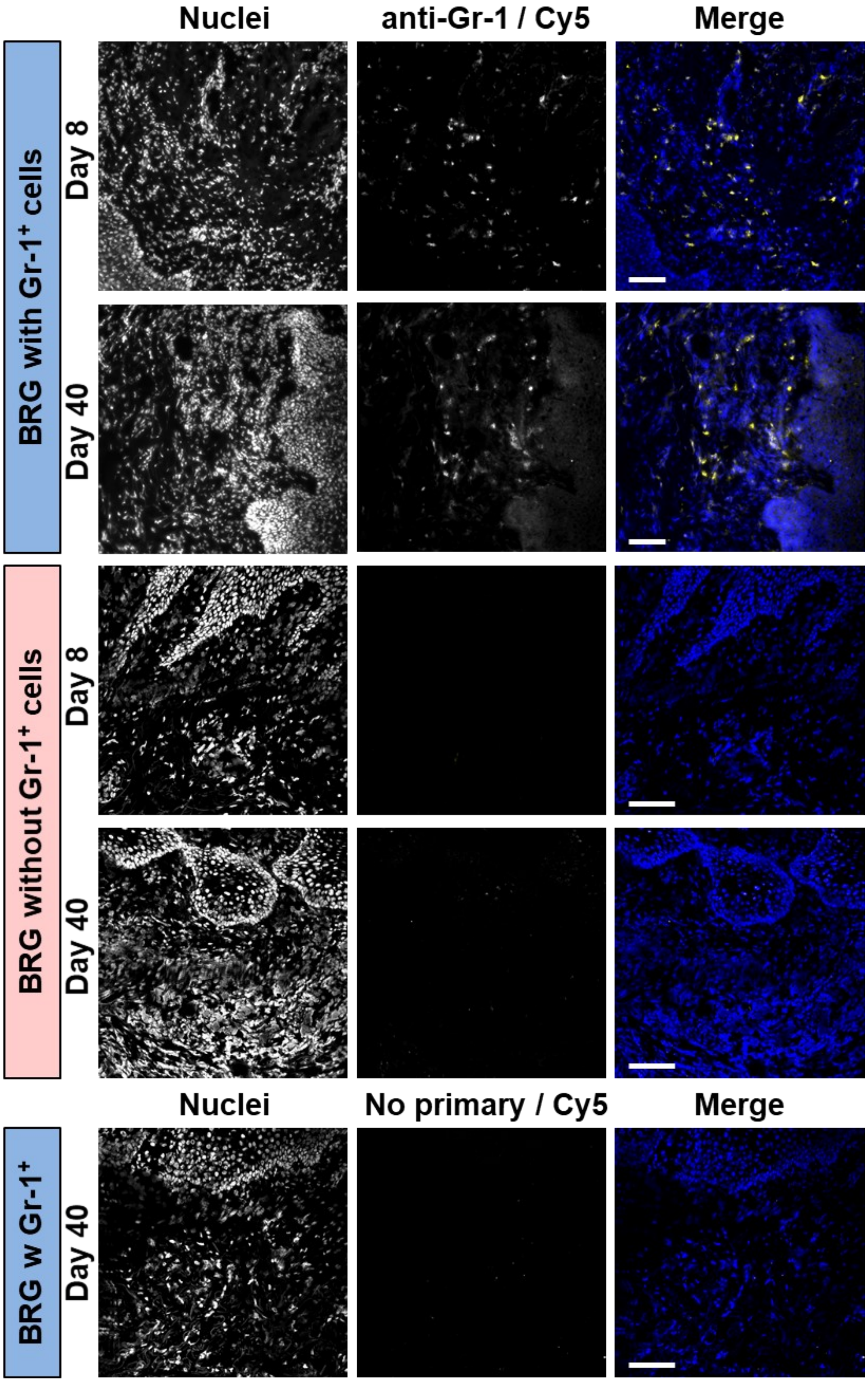

Figure S5 Treatment with anti-Gr-1 antibody depletes Gr-1^{+} cells *in vivo*.| Confocal micrographs of representative human graft sections of animals treated according to the scheme in Fig.4A (day 40; stained as indicated) on the indicated days; scale bars are 100 µm.

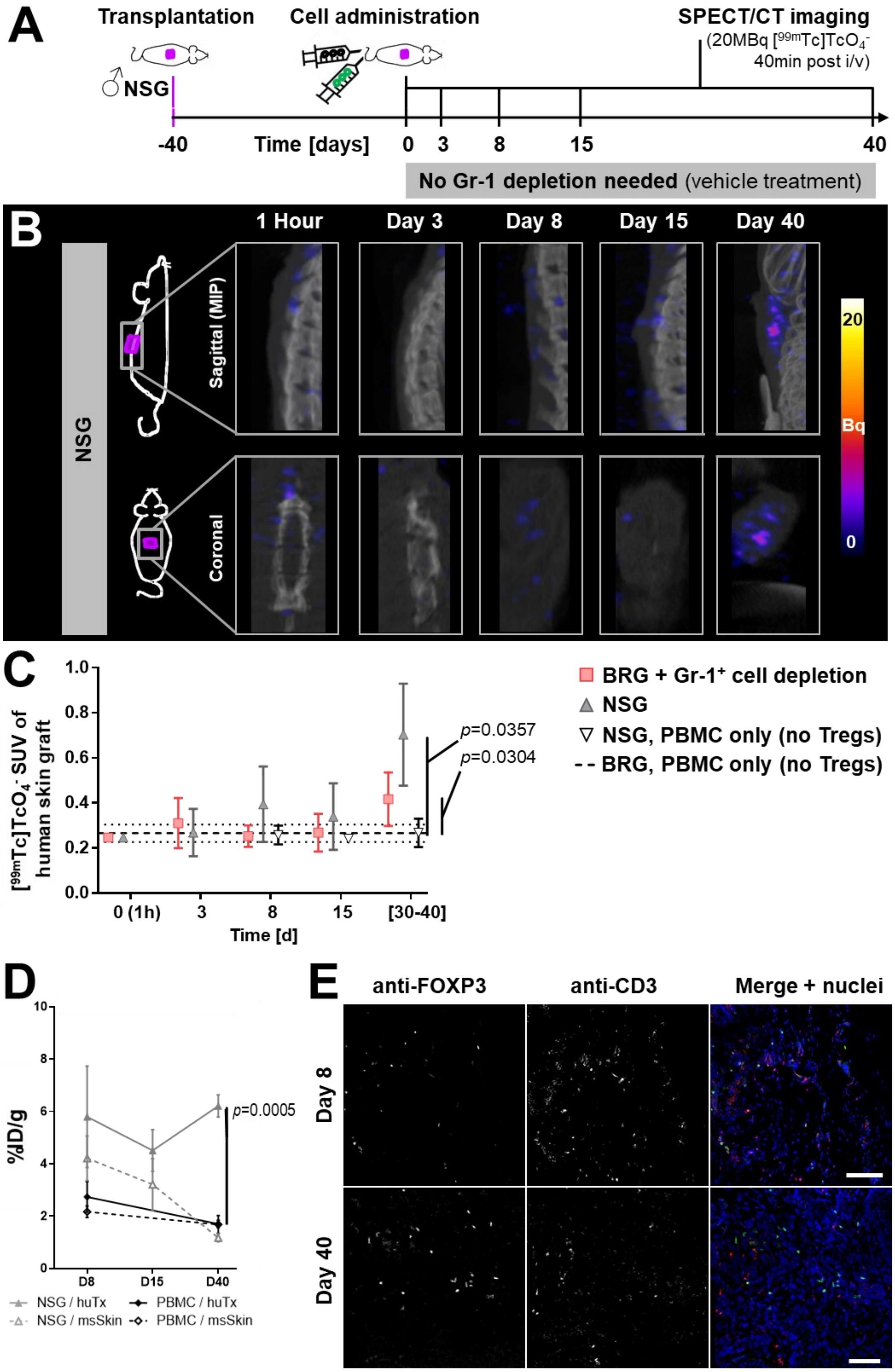

A
Transplantation
Cell administration
SPECT/CT imaging
(20MBq [99mTc]TcO4- 40min post i/v)
♂NSG
-40
Time [days]
0
3
8
15
40
No Gr-1 depletion needed (vehicle treatment)
B
1 Hour
Day 3
Day 8
Day 15
Day 40
NSG
Sagittal (MIP)
Coronal
20
Bq
0
C
[99mTc]TcO4- SUV of human skin graft
Time [d]
0 (1h)
3
8
15
[30-40]
p=0.0357
p=0.0304
BRG + Gr-1+ cell depletion
NSG
NSG, PBMC only (no Tregs)
BRG, PBMC only (no Tregs)
D
%ID/g
p=0.0005
D8
D15
D40
NSG / huTx
NSG / msSkin
PBMC / huTx
PBMC / msSkin
E
anti-FOXP3
anti-CD3
Merge + nuclei
Day 8
Day 40

Figure S6. ***In vivo*** **tracking of NIS-GFP$^+$ Treg arrival at human skin grafts in NSG mice.| (A)** Experimental design: purple=human skin graft, green syringe = 5x10^6 NIS-GFP$^+$ Treg, black syringe = 5x10^6 PBMCs. **(B)** Longitudinal SPECT/CT imaging data of transplant-bearing animals (randomization upon adoptive cell transfer) at indicated time points post adoptive cell transfer. Images acquired and presented as in Fig.4; all images are on the same scale. Radioactivity above background indicates NIS-GFP$^+$ Treg presence. Representative images from the same animal. **(C)** Quantitative SUV analysis of imaging data. Cumulative data of all experiments (n=4); error bars represent SD; multiple t-tests with Holm-Sidak correction for multiple comparisons, p-values shown if < 0.05. For comparison data from Gr-1$^+$ cell-depleted BRG mice are shown (red). **(D)** *Ex vivo* radioactivity analysis of harvested tissues. Mouse skin was harvested from a region above lower spine. Human skin grafts were compared to mouse skin from the same animal using paired t-tests. No Treg/PBMC only control: not significant over all time points (p=0.2526), solid *vs.* dashed black lines; NSG: not significant for early time points (p>0.05) but significant on day 40 (p=0.0020), solid *vs.* dashed gray lines. Human skin grafts from NSG cohorts were compared by ANOVA on day 40 with control mice demonstrating a significant difference (p=0.0005), while mouse tissues did not differ between cohorts (A). Cumulative data; n=4 for days 8 and 40 and n=2 for day 15. **(E)** Immunofluorescence staining of harvested tissues. Indicated times refer to days after adoptive Treg transfer. Representative micrographs of NSG mice that received PBMC and NIS-GFP$^+$ Tregs. CD3 staining is indicative of PBMC-derived human T cells and Tregs, while FOXP3 staining identified human Tregs. Scale bars are 100 µm. Image-based quantification and *ex vivo* confirmation demonstrated a time course of NIS-GFP$^+$ Tregs signals at the transplants comparable to what we had observed for BRG mice with Gr-1$^+$ cell depletion (Fig.4-5).

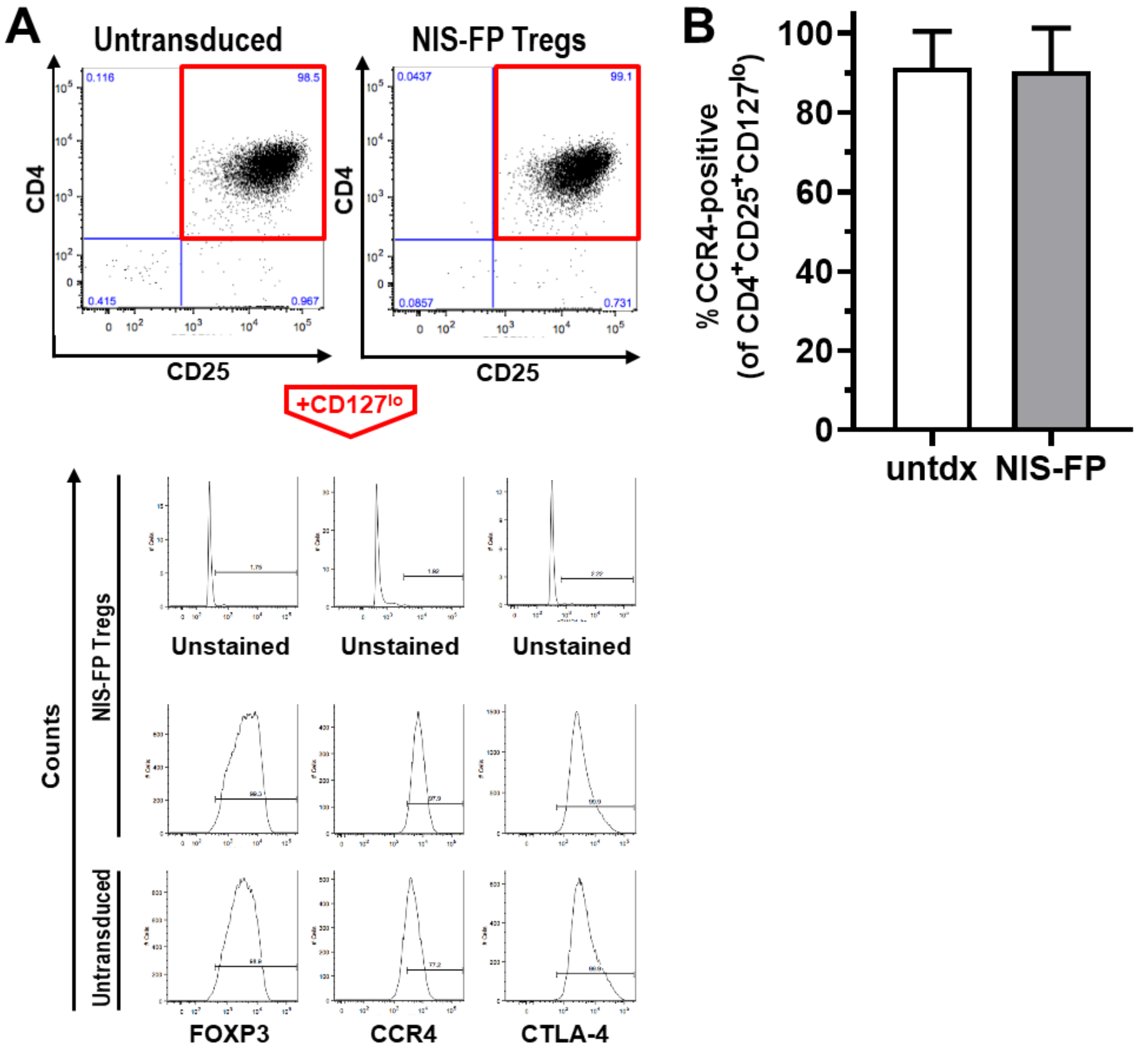

Figure S7. GMP-isolated and *in vitro* expanded Tregs retain their CCR4 expression upon transduction with the reporter gene NIS-FP.| (A) Tregs were isolated from different human donors and expanded *in vitro* for 20 days. $CD4^{+}CD25^{+}CD127^{low}$ Tregs were analyzed for FOXP3, CCR4 and CTLA-4 expression. Representative flow cytometry data are shown as histograms. **(B)** Cumulative data (n=5 individual donors) demonstrating no differences between untransduced and reporter-expressing Tregs (Student's t-test; p=0.8903); error bars represent SD.

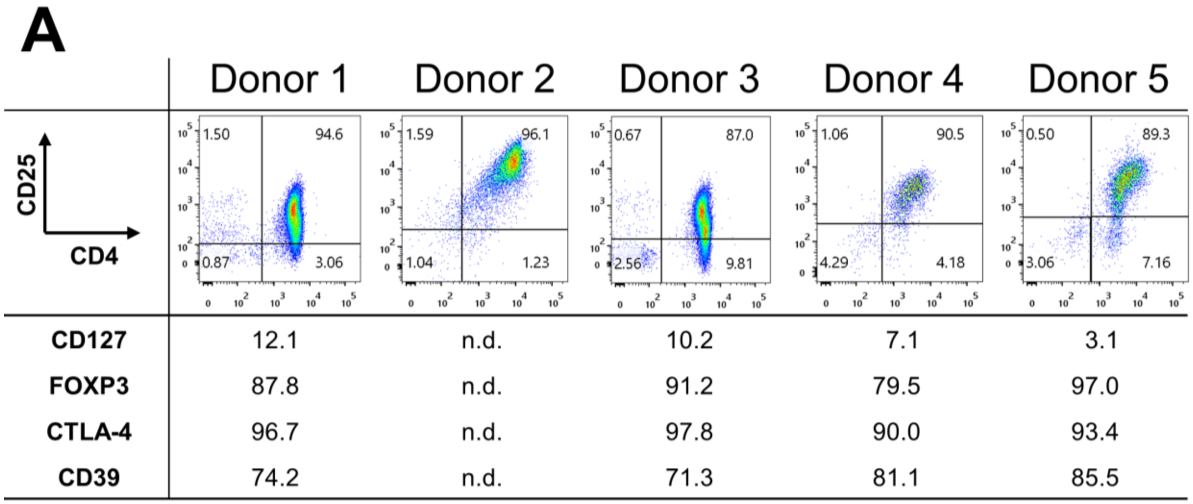

	Donor 1	Donor 2	Donor 3	Donor 4	Donor 5
**CD127**	12.1	n.d.	10.2	7.1	3.1
**FOXP3**	87.8	n.d.	91.2	79.5	97.0
**CTLA-4**	96.7	n.d.	97.8	90.0	93.4
**CD39**	74.2	n.d.	71.3	81.1	85.5

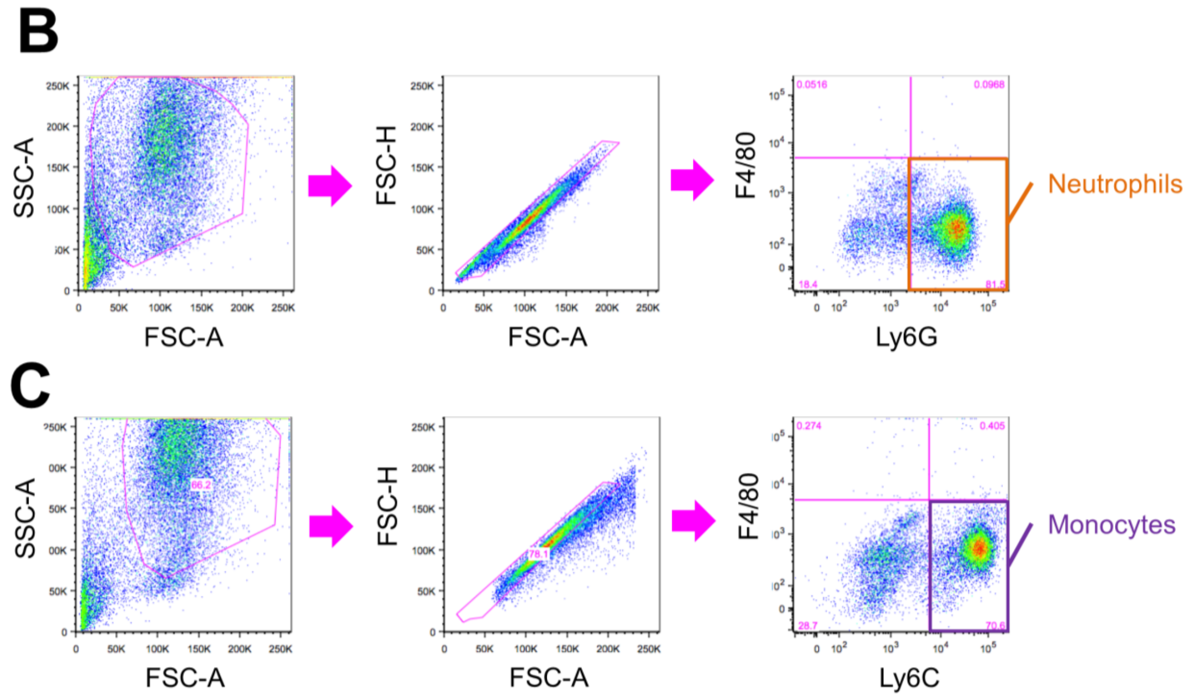

Figure S8. Input quality control data corresponding to purified cells used for experiments shown in Fig.7.| (A) Tregs isolated from individual human donors were analyzed for indicated markers by flow cytometry (numbers indicate percent expressing cells). Enrichment of murine **(B)** neutrophils and **(C)** monocytes.

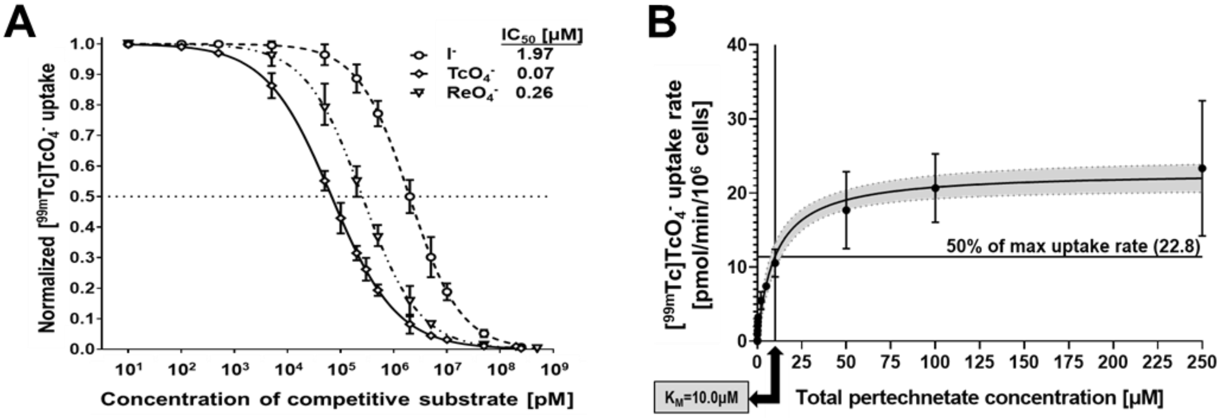

Figure S9. Substrate transport of NIS fused to a fluorescent protein. | (A) NIS anion transport was determined using a substrate competition assay with radioactive $[^{99m}Tc]TcO_4^-$ as the detectable trace anion. Briefly, epithelial breast cancer cells stably expressing NIS-mTagRFP[2] were incubated with 50 kBq/mL $[^{99m}Tc]TcO_4^-$ together with varying concentrations of the indicated anions, which were either non-radioactive I^- and ReO_4^- or the weakly radioactive beta emitter anion $[^{99}Tc]TcO_4^-$ (^{99}Tc radiation did not interfere with γ-emission measurements of ^{99m}Tc). The pertechnetate curve (solid) represents the reference measurement with the data for I^- and ReO_4^- indicating less efficient uptake of these substrates by NIS-mTagRFP compared to pertechnetate. This data is in agreement with data obtained by others using electrophysiological measurements of NIS without C-terminal tail modifications.[4] **(B)** As ^{99m}Tc decays to ^{99}Tc, reliable transport kinetic measurements using the radioactive substrate approach are possible, because the chemical species remains the same despite radioisotope decay and thus permitting constant substrate concentrations throughout the assay. In this way, we used the Michaelis-Menten model to model radiotracer uptake, which was previously shown to be valid for this this process. We determined the Michaelis-Menten constant of NIS-mTagRFP for the transport of the tetrahedral anion pertechnetate to be 10.0 μM (with a 95% confidence interval of [6.4 to 15.6] μM using non-linear regression with least-squares fitting). Pertechnetate is identical in charge and structure, and very similar in its anionic volume compared to ReO_4^-;[5] pertechnetate is also transported by NIS in an electroneutral manner like perrhenate.[6] Our data for the NIS-fusion protein are very close to what was previously reported for the Michaelis-Menten constant of the very similar NIS substrate ReO_4^- using unmodified NIS in an electrophysiological assay (3.5 μM;[7]). All error bars in (A) and (B) represent SD.

While we did not directly compare NIS with a NIS-fluorescent protein fusion in the same cells, we believe our data are sufficiently in agreement with previously reported data on unmodified NIS to suggest that for the purpose of *in vivo* cell tracking the NIS-fluorescent protein fusion reporter is a justified choice. Moreover, the NIS fluorescent protein fusion reporter provides added value by simplifying preclinical experimentation while not negatively impacting on NIS reporter performance. Moreover, the NIS-fluorescent protein reporters have been successfully used for *in vivo* cell tracking in various settings including many cancer cells[1-3, 8, 9], chimeric antigen receptor T-cells[10], and induced pluripotent stem cell-derived hepatocyte-like cells.[8]

<<<End of Supplementary Information>>>
